# Emerging insights into chemistry and therapeutic potentials of functionalized hexahydroquinolines

**DOI:** 10.3389/fchem.2026.1769586

**Published:** 2026-03-05

**Authors:** Gbolahan O. Oduselu, Rhoda O. Olatuyi, Omowunmi O. Fatoki, Damilola S. Bodun, Promise E. Sunday, Wellington Oyibo, Olayinka O. Ajani

**Affiliations:** 1 West African Center for Cell Biology of Infectious Pathogens (WACCBIP), College of Basic and Applied Sciences, University of Ghana, Accra, Ghana; 2 Department of Chemistry, College of Science and Technology, Covenant University, Ota, Nigeria; 3 Department of Pharmaceutical and Medicinal Chemistry, University of Uyo, Uyo, Nigeria; 4 ANDI Center of Excellence for Malaria Diagnosis, College of Medicine, University of Lagos, Lagos, Nigeria

**Keywords:** antiprotozoal agents, drug discovery, heterocyclic compounds, pharmacological potential, quinoline

## Abstract

Quinolines are well-established scaffolds in drug discovery and form the core of many FDA-approved medicines. Among their reduced analogues, hexahydroquinolines (HHQs) have gained increasing attention due to their structural adaptability and broad pharmacological relevance. Research has largely focused on the 1,4,5,6,7,8-hexahydroquinoline framework, although interest in other variants, including 2-oxo derivatives, is steadily growing. This review summarizes recent progress in HHQ chemistry, with emphasis on synthetic strategies and emerging therapeutic applications. Traditional multicomponent reactions such as the Hantzsch, Antaki, and Stankevich methods are discussed alongside more recent green synthetic strategies, including the use of natural and heterogeneous catalysts, catalyst-free protocols, and alternative activation techniques such as ultrasonication. These sustainable protocols often provide improved yields under milder conditions and shorter reaction times. A recurring feature of HHQ synthesis is the preferential formation of 5-oxo derivatives. The mechanistic basis for this structural dominance and the synthetic approaches for the other underexplored HHQ chemotypes are examined. From a biological perspective, HHQs have demonstrated notable activity across several therapeutic areas, including antiprotozoal potential against *Plasmodium falciparum*, *Leishmania* species, and *Toxoplasma gondii*, with several compounds exhibiting dual-stage or transmission-blocking effects. Additional reports also include their usage as modulators of multidrug resistance in cancer cells, antimicrobial, anti-inflammatory, antioxidant, and cardiovascular activities. This review aims to provide a focused and practical reference for researchers engaged in heterocyclic drug design and development involving hexahydroquinoline scaffolds.

## 1 Introduction

Heterocyclic compounds are ring structures that contain at least one atom other than carbon or hydrogen such as oxygen, nitrogen, or sulfur (a heteroatom) within their framework (Srivastava, 2024). They represent one of the most extensive and diverse classes of organic compounds. Examples include furan, pyrrole, thiophene, indole, benzofuran, carbazole, quinoline, isoquinoline, imidazole, oxazole, pyrazole, pyridazine, pyrimidine, purine, and hexahydroquinoline. These compounds exhibit a wide range of pharmacological and biological activities, including antiviral, antioxidant, anticonvulsant, anthelmintic, antipyretic, antihistaminic, herbicidal, antihypertensive, anticancer and antiprotozoal activities (Kabir and Uzzaman, 2022; Srivastava, 2024). Quinoline (benzo[*b*]pyridine) is a particularly important member of the heterocyclic family and serves as a valuable scaffold in the design of novel therapeutic agents (Elebiju et al., 2023).

Modulating the quinoline scaffold has been shown to significantly alter physicochemical and biological properties, supporting its continued exploration in medicinal chemistry (Elebiju et al., 2023). The therapeutic success of quinoline-based drugs prompted extensive investigation of its reduced derivatives, which offer distinct physicochemical and biological profiles. These include 1,2-dihydroquinoline, 1,2,3,4-tetrahydroquinoline, tetrahydroisoquinolines, hexahydroquinolines (1,2,3,4,4a,5-HHQ and 1,4,5,6,7,8-HHQ), octahydroquinoline, and decahydroquinoline (Choudhury et al., 2017; Ranjbar and Khonkarn, 2019; Sridharan et al., 2011). Among these, hexahydroquinolines (HHQs) have emerged as particularly promising scaffolds, combining the pharmacophoric features of quinolines with enhanced conformational flexibility and improved druglike properties (Oduselu et al., 2024; Ranjbar and Khonkarn, 2019). The partially saturated quinoline core tolerates diverse functional substitutions without compromising ring stability, enabling systematic structure–activity relationship (SAR) exploration and fine-tuning of physicochemical parameters such as lipophilicity, solubility, and metabolic stability (Ranjbar and Edraki, 2019; Sridharan et al., 2011).

**FIGURE 1:**
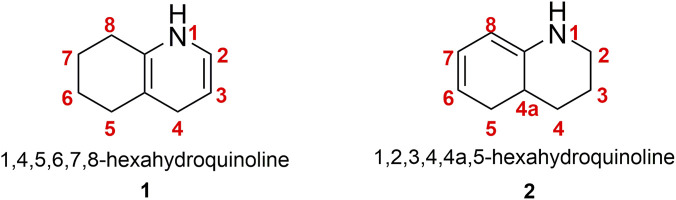
The different tautomeric forms of hexahydroquinolines.

Hexahydroquinolines exist in different structural forms, notably the 1,4,5,6,7,8-hexahydroquinoline (**1**) and the 1,2,3,4,4a,5-hexahydroquinoline (**2**). In compound (**2**), the pyridine ring is fully saturated while the benzene ring contains two unsaturated bonds. In contrast, in compound (1), the pyridine ring is only partially saturated, and the benzene ring is fully saturated (see Figure 1). Among these, the 1,4,5,6,7,8-hexahydroquinoline is the most synthesized form. Hexahydroquinolines (HHQs) are also known to be photochemically unstable. A study by Mielcarek et al. (2002) investigated the effect of various substituents on the phenyl ring on the light sensitivity of HHQ derivatives. The results revealed that the degree of photodegradation depends on both the nature and position of the substituent. Nitro-substituted HHQs were found to be the least stable, whereas compounds bearing alkyl or haloalkyl groups, particularly in the meta position, exhibited the greatest stability under light exposure. In a subsequent study, Mielcarek et al. (2005) further confirmed the formation of single photoproducts for each HHQ derivative examined using HPLC–ESI–MS analysis.

Advances in synthetic chemistry have enabled efficient generation of a wide array of HHQ derivatives, expanding opportunities to explore their biological relevance across multiple therapeutic domains. HHQs are most commonly synthesized through multicomponent reactions, with Hantzsch-type condensations remaining the dominant synthetic route due to their operational simplicity, and ability to rapidly generate molecular diversity (Ranjbar and Khonkarn, 2019). As a consequence, these reactions predominantly afford 5-oxo hexahydroquinoline derivatives, a structural bias arising from the nature of the starting components and the mechanistic pathway of Hantzsch-type condensations. This structural bias has shaped much of the biological literature, with the majority of pharmacological evaluations centered on this chemotype.

More recently, significant efforts have focused on improving the efficiency, sustainability, and scope of HHQ synthesis. Green and alternative synthetic strategies, including heterogeneous nanocatalysis (Andalibi Salem et al., 2021; Dey et al., 2020; Goudarziafsharn et al., 2024) solvent-free and catalyst-free protocols (Devi et al., 2020; Fedoseev et al., 2022; Patil et al., 2017), ultrasound-assisted reactions (Devi et al., 2020; Faidallah and Al-Juaid, 2014), and natural catalysts (Dey et al., 2020; Ghiassi et al., 2019) have enabled higher yields under milder conditions and reduced detrimental environmental impact. Importantly, some of these approaches have also facilitated access to underexplored HHQ chemotypes, such as 2-oxo and 1,2,3,4,4a,5-hexahydroquinolines, which are not readily obtained through classical Hantzsch pathways. This further accelerates the exploration and functionalization of HHQs as drug-like scaffolds.

Beyond their established chemical accessibility, functionalized HHQs have demonstrated a broad spectrum of biological activities. Notably, a number of studies have reported HHQs with promising antiprotozoal activity against *Plasmodium* (Horn et al., 2024; Vanaerschot et al., 2017)*, Leishmania* (Molaei et al., 2023; Oliveira et al., 2024)*, Toxoplasma* (Oliveira et al., 2025; Zahedi et al., 2020), *and Trypanosoma* (Maya et al., 2000) species with activities across multiple targets and life cycle stages. Several HHQ derivatives have demonstrated potent antimalarial activity, including inhibition of *Plasmodium falciparum* blood-stage parasites (Horn et al., 2024) and transmission-blocking effects against mature gametocytes (Oduselu et al., 2024; Vanaerschot et al., 2017). Additional reports describe activity against bacterial and fungal pathogens (Morbale et al., 2015; Mirani Nezhad., 2022), as well as antitubercular effects against *Mycobacterium tuberculosis* (Karad et al., 2015) and antischisostomal activity (Oliveira et al., 2026). However, their anti-infective activity represents only one facet of their broader therapeutic potential. Multiple derivatives have been reported to exhibit anticancer activity (Çetin et al., 2022), particularly through modulation of multidrug resistance mechanisms (Ranjbar and Khonkarn, 2019; Shahraki et al., 2022). Anti-inflammatory effects by COX enzyme inhibition (Zarghi et al., 2014), regulation of cytokine production and complement pathways (Pehli̇Vanlar et al., 2024), alongside antioxidant activity demonstrated through radical scavenging assays, further highlight their pharmacological versatility. Cardiovascular activities, notably calcium-channel antagonism (Gupta and Misra, 2008) have also been documented for selected HHQs.

Despite these advances, the biological and therapeutic relevance of functionalized HHQs remains underexplored relative to other quinoline derivatives. Existing reviews have largely focused on tetrahydroquinolines (Sridharan et al., 2011) or specific HHQ subclasses, such as 5-oxo-hexahydroquinolines (Ranjbar and Khonkarn, 2019) and aminoquinolines (Abdelmoniem et al., 2019), leaving recent developments scattered across the literature. This review provides a comprehensive and up-to-date assessment of functionalized hexahydroquinolines as emerging scaffolds in drug development. We systematically examined the structural diversity of HHQ chemotypes, with emphasis on both prevalent 5-oxo derivatives and underexplored variants (2-oxo and 1,2,3,4,4a,5-HHQs), synthetic strategies ranging from classical multicomponent reactions to modern green chemistry approaches, demonstrated biological activities including mechanistic insights and structure-activity relationships. By consolidating recent advances and identifying future research priorities, this review provides a comprehensive assessment of functionalized hexahydroquinolines, integrating advances in synthetic methodologies, structural diversity, and therapeutic activities, while identifying key gaps and future directions to guide their development as versatile scaffolds in modern drug discovery.

## 2 Synthesis of hexahydroquinoline derivatives

Most HHQ derivatives are synthesized through multicomponent condensation reactions modeled on the classical Hantzsch approach. In general, the formation involves the reaction of a 1,3-dicarbonyl compound, an aromatic aldehyde, and a β-ketoester or aminocrotonate. These components undergo a sequence of Knoevenagel condensation, Michael addition, and intramolecular cyclization steps to generate the hexahydroquinoline ring system (Çetin et al., 2022; Şimşek et al., 2008). Whether carried out through classical reflux conditions, one-pot protocols, or green catalytic systems, the underlying mechanistic pathway is largely consistent across reported methods.

### 2.1 Classical methods

The Hantzsch, Antaki, and Stankevich reactions represent the earliest and most widely studied methods for HHQ synthesis. These approaches, which remain widely used due to their simplicity and versatility, established the mechanistic foundation for multicomponent HHQ formation and provided a framework for structural diversification in modern synthesis.

#### 2.1.1 Hantzsch-type reaction of hexahydroquinoline derivatives

**SCHEME 1:**
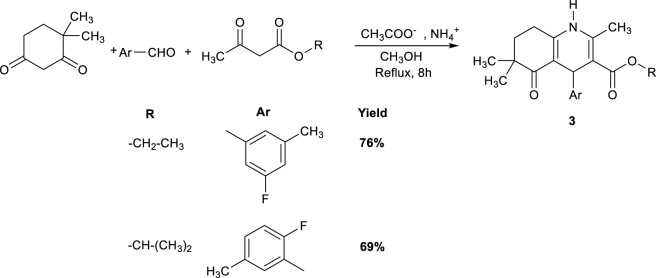
Hantzsch-type synthesis HHQ derivatives.

The classical Hantzsch reaction is normally a one-pot multicomponent condensation method widely used for synthesizing 1,4-dihydropyridines (1,4-DHP) (Zonouz and Abedinpour, 2025). However, it is also commonly adapted, though often modified, for the synthesis of HHQs. In the outcome, the 1,4-DHP scaffold is fused with a cyclohexanone unit to generate the characteristic hexahydroquinoline framework. A representative method (Scheme 1) by Çetin et al. (2022) involved reacting 4,4-dimethylcyclohexane-1,3-dione (1 mmol), an alkyl acetoacetate (1 mmol), a disubstituted benzaldehyde (1 mmol), and ammonium acetate (5 mmol) in 10 mL of methanol to produce HHQ derivatives.

#### 2.1.2 Antaki synthesis of hexahydroquinoline derivatives

**SCHEME 2:**
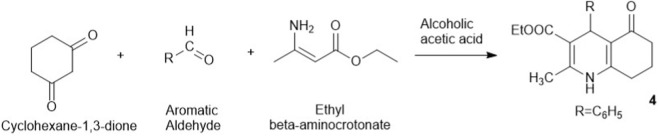
Antaki’s synthesis of HHQ derivatives.

In 1963, Antaki successfully synthesized and isolated substituted 1,4,5,6,7,8-hexahydroquinolines as intermediates with the aim of oxidizing them with chromium trioxide in dilute acetic acid to yield tetrahydroquinolines (Antaki, 1963). The study applied a similar approach to Hantzsch’s condensation, where cyclohexane-1,3-dione, p-nitrobenzaldehyde, and ethyl β-aminocrotonate were reacted (Scheme 2). The reaction was conducted using equimolar amounts of the reactants, and the mixture was heated with stirring at the boiling point in a solution of ethanol (180 mL) and glacial acetic acid (60 mL) for 1 h.

#### 2.1.3 Stankevich synthesis of hexahydroquinoline derivatives and modifications

**SCHEME 3:**
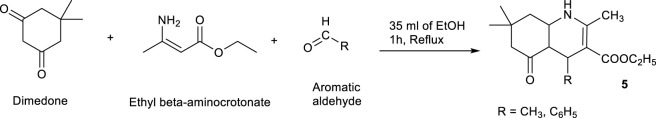
Stankevich’s synthesis of HHQ derivatives.

In a related study following a similar reaction to Antaki’s method, Stankevich et al. (1975) synthesized substituted HHQs by reacting dimedone, ethyl β-aminocrotonate, and paraformaldehyde. The reaction mixture was refluxed in 35 mL of ethanol for 1 h, during which the solution gradually turned dark yellow (Scheme 3). Upon completion, the reaction was cooled and diluted with water, leading to the precipitation of a pale-yellow crystalline product. The yield obtained was 1.45 g (37.8%), with a melting point of 172 °C–173 °C (from aqueous ethanol).

Similarly, Abou-Gharbia (1986) reported the synthesis of corresponding hexahydroquinoline carboxylic esters by refluxing two heterocyclic aldehydes (N-methylimidazole-2-carboxaldehyde and thiazole-2- carboxaldehyde) with equimolar quantities of 1,3-cyclohexanedione (dimedone) and ethyl-3-aminocrotonate in ethanol (Scheme 3), affording products in 37% and 72% yields.

**SCHEME 4:**
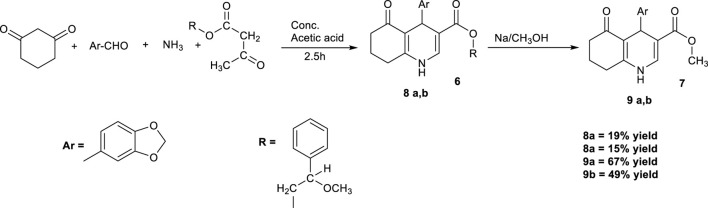
Synthesis of HHQ derivatives via the modified Stankevich’s synthesis.

Rose and Drager (1992) modified the procedure in another reaction following the same mechanism by replacing ethanol with a mixture of 5 mL of ammonia solution and 5 mL of concentrated acetic acid (Scheme 4). Ammonia served as the nitrogen source for constructing the pyridine ring. As the third reactant, an acetoacetate ester containing an optically active alcohol was used as a chiral auxiliary reagent. The reaction mixture was heated to boiling for 2.5 h. Upon cooling, the crude precipitate that was formed was filtered and recrystallized from methanol to yield colorless, needle-like crystals with a melting point of 234 °C. However, the overall yield was low (19% and 15%). Further treatment with metallic sodium in methanol successfully removed the bulky chiral group, affording the desired compounds 9a and 9b (Scheme 4).

#### 2.1.4 One-pot three-component synthesis of hexahydroquinoline-3-carboxamides

**SCHEME 5:**
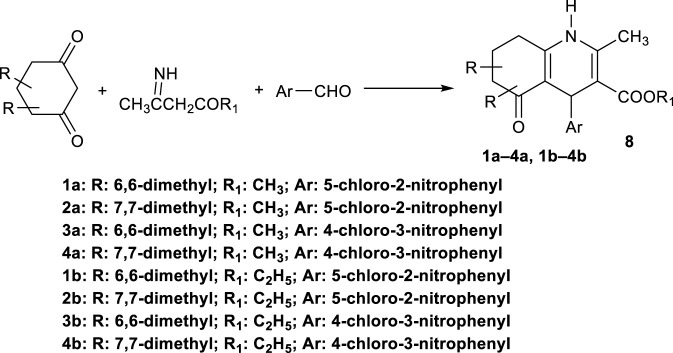
One-pot three-component synthesis of HHQ derivatives.

Şimşek et al., in 2008 generated ester derivatives of HHQs (**1a**–**4a** and **1b**–**4b**) via a modified one-pot multicomponent Hantzsch’s reaction. They were prepared by reacting 4,4- or 5,5-dimethyl-1,3-cyclohexanedione with the appropriate aromatic aldehyde and methyl (or ethyl) aminocrotonate (Scheme 5).

### 2.2 Green synthesis approach

Although the classical methods are foundational, they are often limited by low yields, long reaction times, reliance on volatile organic solvents, and generally harsh reaction conditions. Due to this, there has been a shift in focus to develop more efficient alternative methods of HHQ synthesis that use cleaner energy inputs and less hazardous media, along with catalysts that are reusable, biodegradable, or easier to handle in recent works. Approaches such as ultrasound- and microwave-assisted synthesis, solvent-free or grinding methods, ionic liquids, reusable nanocatalysts, organocatalysts, and metal–organic framework–based catalysts have all been explored as greener and more sustainable routes (Ranjbar and Khonkarn, 2019).

#### 2.2.1 Catalyzed green methods

##### 2.2.1.1 Green synthesis of hexahydroquinolines using water-dispersed magnetic nanoparticles (γ- Fe_2_O_3_) as catalyst

**SCHEME 6:**
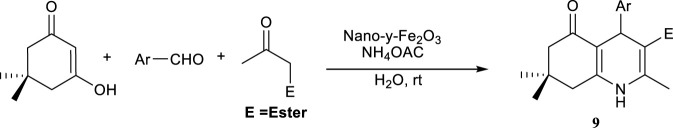
Green synthesis of HHQs using water-dispersed magnetic nanoparticles (γ- Fe_2_O_3_).

In 2013, Rostamnia and co-workers reported the use of reusable, cost-effective, and non-toxic magnetic nanoparticles (γ-Fe_2_O_3_) dispersed in water as an ecofriendly catalyst for the preparation of hexahydroquinolines (HHQs). This method provided improved yields, averaging 92%, outperforming conventional reactions (Scheme 6). The reaction involved a one-pot, multicomponent condensation of an aldehyde (1 mmol), dimedone (1 mmol), a β-dicarbonyl compound (1.2 mmol), and ammonium acetate (1.5 mmol), catalyzed by 10 mol% γ- Fe_2_O_3_ nanoparticles in 3 mL of water.

##### 2.2.1.2 Nanomagnetic Cu(II) catalyst for solvent-free synthesis of hexahydroquinoline derivatives

**SCHEME 7:**
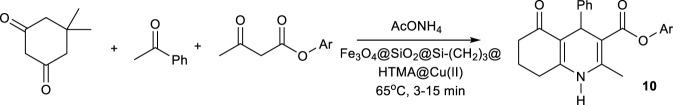
Synthesis of HHQ derivatives using magnetic Cu(II) nanocatalyst.

Andalibi Salem et al. (2021) used a nanomagnetic Cu(II) catalyst derived from a nano-Fe_3_O_4_ core to improve efficiency in synthesis of HHQ derivatives. It functioned as a rigid heterogeneous catalyst under ecofriendly, non-solvent conditions. The catalyst, Fe_3_O_4_@SiO_2_@Si-(CH_2_)_3_@HMTA@Cu(II), was synthesized through a multi-step process beginning from nano-Fe_3_O_4_. Under optimized conditions, the catalyst facilitated the synthesis of various HHQ derivatives in moderate to high yields (50%–96%) within limited reaction times of just 3–15 min. After completion of the reaction (Scheme 7), the mixture was cooled, and ethyl acetate was added. Due to the catalyst’s insolubility in ethyl acetate, it could be readily separated from the reaction mixture. The study also compared the effects of various solvents and solvent-free conditions on both reaction time and yield. The solvent-free reaction was completed in just 6 min with a yield of 85%, whereas reactions performed in the presence of solvents showed lower yields and required longer times.

##### 2.2.1.3 Hexahydroquinoline synthesis using natural catalysts

**SCHEME 8:**
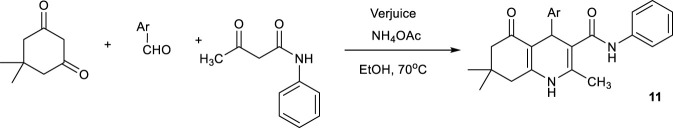
Verjuice-catalyzed synthesis of hexahydroquinoline-3-carboxamide derivatives.

Verjuice, the acidic juice of unripe grapes, has been recognized as a natural, biocompatible catalyst. Its mild acidity effectively promotes condensation reactions, offering a green alternative to conventional mineral acids in the synthesis of heterocyclic compounds. A study by Ghiassi et al. (2019) explored the application of unripe grape juice (verjuice, pH 2.9) as a natural and biocompatible catalyst for the green one-pot synthesis of hexahydroquinoline-3-carboxamide derivatives through a four-component reaction of aryl aldehydes, dimedone, acetoacetanilide, and ammonium acetate. The reactions were carried out in ethanol at 70 °C using 10 drops of verjuice, with the model reaction yielding 95% of the desired product in 20 min (Scheme 8). A variety of derivatives were synthesized with yields ranging from 86% to 96%. Compared to traditional methods, the verjuice-catalyzed protocol offered milder conditions, shorter reaction times, and higher yields.

##### 2.2.1.4 imidazole in green synthesis of hexahydroquinoline derivatives

**SCHEME 9:**
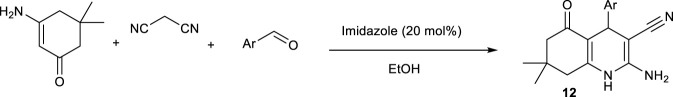
Green Synthesis of HHQs with imidazole as catalyst.

To determine whether imidazole acts as a catalyst in multicomponent reactions involving aromatic aldehydes, 3-aminodimedone, and malononitrile for HHQ synthesis, Moshtaghi et al. (2016) presented and compared two syntheses routes of HHQ derivatives. In the first route (Scheme 9), a mixture of 3-nitrobenzaldehyde (150 mg, 1 mmol), malononitrile (70 mg, 1 mmol), and 3-amino-5,5-dimethyl-2-cyclohexen-1-one (140 mg, 1 mmol) was refluxed in ethanol (5 mL) for 22 h. Upon completion (as monitored by TLC), the reaction mixture was cooled, and the resulting precipitate was filtered and recrystallized from hot ethanol. This yielded 2-amino-7,7-dimethyl-5-oxo-4-(3-nitrophenyl)-1,4,5,6,7,8-hexahydroquinoline-3-carbonitrile as yellow crystals (246 mg, 76%). While using the second method, the same reactants were used with the addition of imidazole (10 mg, 0.2 mmol) as a catalyst. The reaction was completed within 45 min under reflux in ethanol (5 mL). The product was isolated following the same workup and recrystallization process.

##### 2.2.1.5 Cellulose-based magnetic bionanocomposite (γ- Fe_2_O_3_ Cu@cellulose) as a green recyclable catalyst for hexahydroquinoline synthesis

**SCHEME 10:**
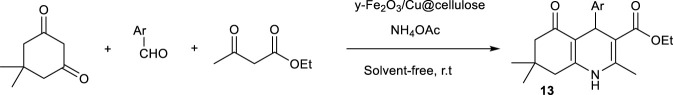
Synthesis of HHQs using cellulose-based magnetic bionanocomposite.

Maleki (2017) introduced a new green catalyst made from a cellulose-based magnetic bionanocomposite called γ-Fe_2_O_3_/Cu@cellulose.This material was tested as a solid, reusable catalyst for one-pot reactions to make Hantzsch 1,4-dihydropyridines and HHQ derivatives. The study then optimized the reaction conditions and found that performing the reaction without any solvent gave the best results (Scheme 10). Among the tested catalyst amounts, 3 mg was found to be optimal, however using more did not improve the yield.

The catalyst worked well with different aromatic aldehydes, giving high product yields between 80% and 98%, with short reaction times of 9–35 min. It was readily isolated from the reaction using a magnet and reused for at least five cycles without losing efficiency. After repeated use, tests showed that the copper content stayed around 10%, confirming there was no significant leaching and that the catalyst remained stable.

##### 2.2.1.6 Schiff-based palladium nanocatalyst for green synthesis of hexahydroquinolines

**SCHEME 11:**
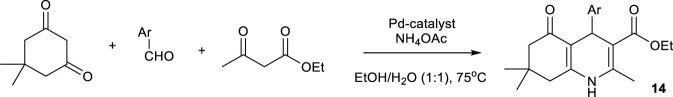
Green synthesis of HHQs using Fe_3_O_4_ supported Schiff-base palladium.

A study carried out by Ghorbani et al. (2015) reported the synthesis of a new Schiff-base palladium complex supported on Fe_3_O_4_ magnetic nanoparticles and evaluated its use as a heterogeneous catalyst for the preparation of HHQs. The Fe_3_O_4_-supported Schiff-base Pd complex, referred to as catalyst F, was examined in a one-pot, multicomponent condensation reaction (Scheme 11).

Reaction optimization was carried out and the best results were achieved using 20 mg of catalyst F in a 1:1 ethanol-water mixture at 75 °C, with equimolar amounts of aldehyde, dimedone, and ethyl acetoacetate. Under these conditions, a 94% yield was obtained within 9 min. In contrast, reactions carried out without a catalyst or with a metal-free catalyst gave lower yields or required significantly longer times. The catalyst demonstrated broad applicability in synthesizing a range of HHQ derivatives using different aldehydes, consistently affording high yields between 83% and 94% within short reaction times of 6–12 min. The recyclability of catalyst F was also assessed in the model reaction. The catalyst was magnetically recovered, washed, dried, and reused for six successive cycles without any notable loss in catalytic performance.

##### 2.2.1.7 Green synthesis of hexahydroquinolines using sulfonated rice husk (SRH) as a recyclable catalyst

**SCHEME 12:**
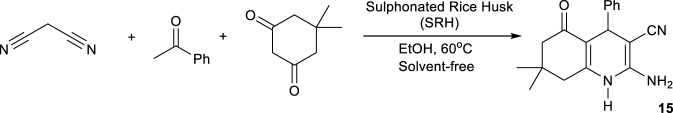
Green synthesis of HHQs using sulphonated rice husk as a recyclable catalyst.

An efficient, simple, and environmentally friendly method for synthesizing bioactive HHQ derivatives and hexahydroacridine-1,8-diones using a new biodegradable solid catalyst called sulfonated rice husk (SRH) was reported by Dey et al., in 2020. According to the study, this method offered several key advantages, such as easy operation, greener reaction conditions, and the ability to reuse the catalyst multiple times. The study explained that carbon-based sulfonated catalysts like SRH are promising because they contain distinct acidic groups and have a large carbon structure that supports the reaction. The synthesis gave excellent yields, with results reported as high as 98%. In particular, they found that under optimized conditions, HHQ derivatives could be produced in 96% yield within just 20 min at 60 °C, using 60 mg of SRH under solvent-free conditions (Scheme 12). The catalyst also showed strong reusability, as it retained its acidity and effectiveness through seven consecutive reaction cycles without a noticeable drop in performance.

##### 2.2.1.8 Eggshell catalyst synthesis of hexahydroquinoline

**SCHEME 13:**
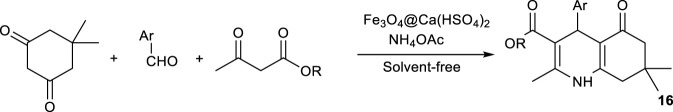
Eggshell-catalyzed synthesis of HHQs.

Eggshells, which are rich in calcium carbonate and readily available as biowaste, can be transformed into efficient heterogeneous catalysts with good recyclability and low environmental impact. Akbarpoor et al. (2020) converted waste eggshells into a valuable nanomagnetic solid acid catalyst, Fe_3_O_4_@Ca(HSO_4_)_2_ and evaluated its efficiency in the synthesis of HHQ derivatives. Optimization studies identified the optimal conditions as 0.05 g of the catalyst at 71 °C under non-solvent conditions. Under these conditions, various HHQ derivatives were synthesized in short reaction times of 3–9 min, with yields ranging from 68% to 98% (Scheme 13). The catalyst was isolated after the reaction by dissolving the product in hot ethanol and using an external magnet for separation. Reusability tests demonstrated stable performance over three cycles, yielding 87%, 85%, and 79%, respectively.

**SCHEME 14:**
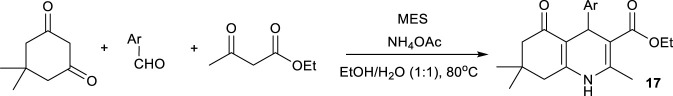
Synthesis of HHQs catalyzed by Modified Eggshells (MES).

A similar study by Morbale et al. (2015) investigated the use of modified eggshells (MES) instead. Optimization of the model reaction established the optimal conditions as 10 wt.% MES in a 1:1 ethanol–water solvent system at 80 °C for 45 min, affording a 96% yield. Under these conditions, various HHQ derivatives were synthesized in good to excellent yields, ranging from 83% to 91%, with reaction times between 45 and 70 min (Scheme 14).

##### 2.2.1.9 Peanut shell–derived nanocatalyst (porous carbon/Fe_3_O_4_ nanocomposite) for solvent-free hexahydroquinoline synthesis

**SCHEME 15:**
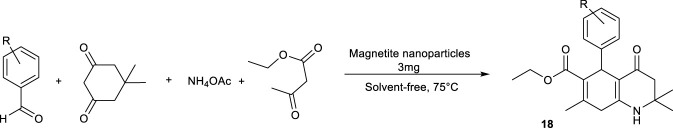
Synthesis of HHQs catalyzed by peanut shell–derived nanocatalyst.

Goudarziafshar et al. (2024) developed a solvent-free multicomponent synthesis of hexahydroquinoline derivatives using a peanut shell–derived porous carbon/Fe_3_O_4_ nanocomposite as a heterogeneous catalyst that was magnetically recoverable and reusable. The catalyst was prepared by high-temperature pyrolysis of peanut shell biomass at 600 °C for 4 h then subsequent combination with Fe_3_O_4_ nanoparticles in ratio 3:1. Fourteen HHQs were synthesized from aryl aldehydes, dimedone, ethyl acetoacetate, and ammonium acetate at 75 °C. The reaction (Scheme 15) proceeded efficiently within 6–22 min, affording high yields (81%–96%) using only 3 mg of catalyst.

##### 2.2.1.10 Catalyzed hexahydroquinolines synthesis optimized using central composite design (CCD)

**SCHEME 16:**
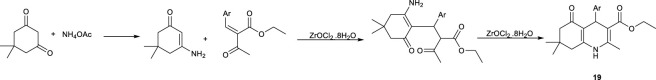
Synthesis of HHQs catalyzed by zirconyl chloride octahydrate.

Khazaei et al. (2015) retained the classical Hantzsch four-component route to synthesize HHQs but focused on making the process statistically optimized and ultrafast. They used commercial ZrOCl_2_·8H_2_O as a simple Lewis acid catalyst, exploiting Zr^4+^ mediated carbonyl activation to accelerate the key Knoevenagel and Michael steps. Rather than empirical trial-and-error, the reaction was optimized using Design of Experiments (Central Composite Design) to model the combined influence of temperature and catalyst loading on yield. The model predicted an optimum at 83.75 °C and 0.15M catalyst, which was experimentally validated at ∼85 °C, showing close agreement between predicted and observed yields. Under these conditions, hexahydroquinolines formed in 40–180 s with yields between 79%–98% (Scheme 16). Nonetheless, yield loss was observed at higher temperatures and the study further demonstrated that this was due to thermal dehydration and structural changes of the catalyst.

Because the conventional approaches to HHQ synthesis commonly deliver high yields at the expense of extended reaction times and cumbersome catalyst recovery, Khazaei (2017) further engineered a magnetically separable copper catalyst by immobilizing a picolinaldehyde–melamine Cu complex onto an Fe_3_O_4_@SiO_2_ support as opposed to ZrOCl_2_·8H_2_O used in their previous study. The catalyst architecture was designed to combine strong Lewis acid activation with facile magnetic retrieval. Response surface methodology using a face-centred central composite design was also applied and the model predicted an optimum condition at 0.07 g catalyst and 87.6 °C, which was confirmed experimentally with minimal deviation. The hexahydroquinoline derivatives were obtained under solvent-free conditions within 2–17 min, affording 71%–92% isolated yields, with the benchmark reaction reaching 91% yield in 6 min. The catalyst was readily recovered by magnetic separation and reused over multiple cycles.

In 2018, Khazaei et al. developed another magnetically recoverable Brønsted-acid ionic-liquid catalyst by anchoring high-density SO_3_H groups onto a melamine-functionalized Fe_3_O_4_ support for the solvent-free Hantzsch synthesis of hexahydroquinolines. The fitted model identified 65 °C and 0.04 g catalyst as optimal. Under these conditions, HHQ derivatives were formed within 4–11 min in 48%–90% isolated yields.

##### 2.2.1.11 Green synthesis of hexahydroquinolines using nano-SiO_2_/taurine bio-derived catalyst

**SCHEME 17:**
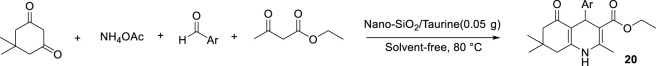
Synthesis of HHQs using Nano-SiO_2_/Taurine bio-derived catalyst.

Sharifabad et al. (2022) reported the development of a recoverable and reusable taurine-based solid acid catalyst (Nano-SiO_2_/Taurine) and evaluated its performance in the classical Hantzsch synthesis of hexahydroquinolines (Scheme 17). Taurine was immobilized on nano-silica to generate a stable, Brønsted-acidic, heterogeneous catalyst, which was extensively characterized to confirm successful grafting and thermal stability. Optimization showed that 0.05 g catalyst at ∼80 °C–85 °C provided the best results. Under these conditions, a range of HHQs were obtained in 10–35 min with high isolated yields (≈86–96%).

In 2024, Loukhmi et al. also developed a renewable nanostructured diphosphate catalyst (Na_2_CaP_2_O_7_), via a solvent-free dry method, characterized by FTIR, XRD, SEM, and TEM, to promote the condensation of aryl aldehydes, malononitrile or ethyl cyanoacetate, dimedone, and ammonium acetate in HHQ synthesis. The nano pyrophosphate efficiently enabled reaction completion within 10 min under mild conditions, delivering high yields (62%–97%) in ethanol at 80 °C.

#### 2.2.2 Catalyst-free green methods

##### 2.2.2.1 Catalyst-free green synthesis of hexahydroquinolines

**SCHEME 18:**
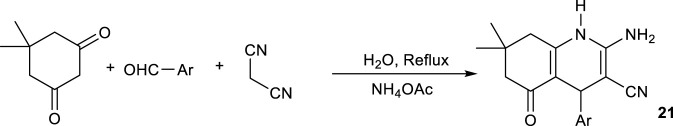
Catalyst-free green synthesis of HHQs.

Guided by green chemistry principles, recent catalyst-free strategies for HHQ synthesis emphasize reduced energy input and simplified purification processes that avoid chromatographic techniques. In some cases, aqueous media are employed as environmentally benign reaction solvents, contributing to more sustainable HHQ synthesis. Patil et al. (2017) synthesized HHQs using dimedone, ammonium acetate, aryl aldehydes, and malononitrile in aqueous media without employing any external catalyst. In this method, excess ammonium acetate is used as both a reagent and an internal catalyst. Optimization studies were performed using dimedone, ammonium acetate, 3-trifluoromethylbenzaldehyde, and malononitrile as model substrates (Scheme 18). Initial attempts without excess ammonium acetate yielded only trace product (∼30%) after 7 h of reflux in water. However, the use of 2 mmol of excess ammonium acetate at reflux significantly improved the outcome, delivering an 89% yield within just 1 h. Among the solvents tested, including ethanol, methanol, isopropanol, DMF, and DMSO, water proved to be the most effective, further showing the ecofriendly nature of this method catalyst.

##### 2.2.2.2 Catalyst-free ultrasonic synthesis of 2-methyl-5-oxo-hexahydroquinoline-3-carboxylate derivatives

**SCHEME 19:**
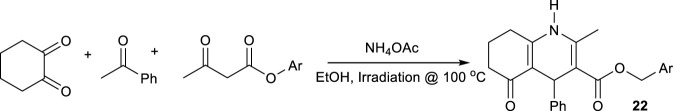
Ultrasound -assisted, catalyst-free synthesis of 2-Methyl-5-oxo-hexahydroquinoline-3-carboxylate derivatives.

To override the use of a catalyst in HHQ synthesis, Devi and colleagues (2020) developed a fast, sustainable, and ecofriendly method for the non-catalyzed synthesis of substituted 2-methyl-5-oxo-hexahydroquinoline-3-carboxylate aided by ultrasonication. The protocol employed a one-pot, multicomponent reaction involving various benzaldehydes, 1,3-cyclohexanedione, benzyl acetoacetate, and ammonium acetate, using ethanol as the solvent and ultrasonic irradiation at room temperature (Scheme 19). Notably, the reaction proceeded efficiently without the need for any catalyst. This method enabled the successful preparation of novel 2-methyl-5-oxo-hexahydroquinoline-3-carboxylate derivatives, achieving excellent yields ranging from 92% to 98% within 10 min. Optimization studies confirmed that ultrasonic irradiation in ethanol offered the best performance, whereas non-polar and aprotic polar solvents were less effective, and polar protic solvents such as isopropanol and methanol led to longer reaction times and lower yields compared to ethanol.

The reported developments from classical to green synthetic methodologies for hexahydroquinoline derivatives, including catalyzed and catalyst-free protocols, are summarized in Table 1.

**TABLE 1:** Synthetic strategies for structurally dominant hexahydroquinoline derivatives.

S/N	Category	Method/Catalyst	Key reagents	Conditions	Time	Yield	Unique advantage
1	Classical (Çetin et al., 2022)	Hantzsch-type	4,4-dimethylcyclohexane-1,3-dione, acetoacetate, disubstituted benzalaldehyde, ammonium acetate	Refluxed in methanol, one-pot	8 h	69%–76%	Simple multicomponent foundation method
2	Classical (Antaki, 1963)	Antaki synthesis (modified Hantzsch-type)	Cyclohexane-1,3-dione, ethyl β-aminocrotonate, nitrobenzaldehyde	EtOH + acetic acid, reflux	1 h	–	First isolated HHQs as intermediates
3	Classical (Stankevich et al., 1975)	Stankevich Synthesis (modified Hantzsch-type)	Dimedone, ethyl β-aminocrotonate, paraformaldehyde	Reflux in EtOH	1 h	37.8%	Straightforward, forms crystalline HHQs
4	Classical (modified) Abou-Gharbia (1986)	Modified Stankevich Synthesis	Dimedone, ethyl 3-aminocrotonate, heterocyclic aldehydes (N-methylimidazole-2-carbox- aldehyde and thiazole-2- carboxaldehyde)	Reflux in EtOH	12 h	37%–72%	Enhances structural diversity
5	Classical (modified) Rose and Drager (1992)	Modified Stankevich Synthesis	Dimedone, acetoacetate ester, benzaldehyde	Ammonia, conc. acetic acid heat, metallic sodium in methanol	2.5 h	15%–19%	Produces chiral HHQ precursors, the bulky chiral groups were removed with metallic sodium in methanol
6	One-Pot/MCR (Şimşek et al., 2008)	Modified Hantzsch-type	4,4- or 5,5-dimethyl-1,3-cyclohexanedione aldehyde, methyl (or ethyl) aminocrotonate, aromatic aldehyde	One-pot MCR, refluxed in methanol	4 h	63%–81%	Fast one-pot multicomponent carboxamide formation
7	Green (Catalyzed) Rostamnia et al. (2013)	Water dispersed γ-Fe_2_O_3_ nanoparticles	Aromatic aldehyde, dimedone, β-dicarbonyl compound, ammonium acetate	Water, 10 mol% γ- Fe_2_O_3_catalyst	2.5–3 h	90%–96%	Water-based, reusable catalyst
8	Green (Catalyzed) Andalibi Salem et al. (2021)	Cu(II) nanomagnetic catalyst, (Fe_3_O_4_@SiO_2_@Si-(CH_2_)_3_@HMTA@Cu(II))	Aldehyde, dimedone, acetoacetate	Solvent-free	3–15min	50%–96%	Very fast, high efficiency, catalyst separable from reaction mixture with ethyl acetate
9	Green (Natural Catalyst) Ghiassi et al. (2019)	Verjuice (unripe grape juice)	Aryl aldehyde, dimedone, acetoacetanilide, ammonium acetate	EtOH, 70 °C, natural acid catalyst	20 min	86%–96%	Biocompatible acidic catalyst, milder conditions
10	Green (Catalyzed) Moshtaghi et al. (2016)	Imidazole catalyst	Nitrobenzaldehyde, malononitrile, 3-aminodimedone	EtOH reflux + imidazole	45 min	76%	Cuts reaction time drastically relative to the non-catalyzed synthesis
11	Green (Catalyzed, statistically optimized) (Khazaei et al., 2015)	ZrOCl_2_·8H_2_O Lewis acid catalyst with CCD optimization	Aldehyde, dimedone, β-dicarbonyl, ammonium acetate	Solvent-free, 85 °C, 0.15 M catalyst	40–180 s	79%–98%	Ultrafast HHQ formation guided by Central Composite Design
12	Green (Catalyzed, statistically optimized) Khazaei (2017)	Fe_3_O_4_@SiO_2_-supported Cu complex with CCD optimization	Aldehyde, dimedone, β-dicarbonyl, ammonium acetate	Solvent-free, 88 °C	2–17 min	71%–92%	Strong Lewis acid activation with magnetic recyclability
13	Green (Catalyzed, statistically optimized) Khazaei et al. (2018)	SO_3_H-functionalized - Fe_3_O_4_supported Brønsted acidic ionic liquid, with CCD optimization	Aldehyde, dimedone, β-dicarbonyl, ammonium acetate	Solvent-free, 65 °C	4–11 min	48%–90%	Recyclable, magnetic recovery
14	Green (Bionanocomposite) Maleki (2017)	γ-Fe_2_O_3_/Cu@cellulose	Aromatic Aldehyde, dimedone, ethyl acetoacetate	Solvent-free, 3 mg catalyst	9–35 min	80%–98%	Highly reusable catalyst
15	Green (Metal Nanocatalyst) Ghorbani et al. (2015)	Pd-Schiff base on Fe_3_O_4_	Aromatic Aldehyde, dimedone, ethyl acetoacetate	EtOH:H_2_O (1:1), 75 °C	6–12 min	83%–94%	High turnover, recyclable (magnetically recovered), short reaction time
15	Green (Bio-derived catalyst) (Sharifabad et al., 2022)	Nano-SiO_2_/Taurine solid acid catalyst	Aldehyde, dimedone, acetoacetate, ammonium acetate	Solvent-free, 80 °C–85 °C	10–35 min	∼86–96%	Bio-derived taurine catalyst; stable, reusable
16	Green (Nanostructured Inorganic catalyst) Loukhmi et al. (2024)	Nano-Na_2_CaP_2_O_7_ diphosphate catalyst	Aldehydes, malononitrile or ethyl cyanoacetate, dimedone, ammonium acetate	EtOH, 80 °C	≤10 min	62%–97%	Rapid one-pot synthesis using recyclable nanopyrophosphate catalyst
17	Green (Biowaste Catalyst) Dey et al. (2020)	Sulfonated rice husk (SRH)	Malononitrile, dimedone, 1-phenylethanone	Solvent-free, 70 °C, 60 mg SRH	20 min	Up to 96%	Cheap, biodegradable, highly reusable
18	Green (Biowaste Catalyst) Akbarpoor et al. (2020)	Eggshell-derived Fe_3_O_4_@Ca(HSO_4_)_2_catalyst	Aromatic aldehyde, dimedone, acetoacetate	Solvent-free, 71 °C	3–9 min	68%–98%	Converts waste to high-performing catalyst
19	Green (Biowaste Catalyst) Morbale et al. (2015)	Modified eggshells (MES)	Aromatic aldehyde, dimedone, acetoacetate	EtOH:H_2_O (1:1), 80 °C	45–70 min	83%–91%	Cheap, sustainable catalyst
20	Green (Biowaste Nanocatalyst) (Goudarziafshar et al., 2024)	Peanut shell–derived porous carbon/Fe_3_O_4_ nanocomposite	Aryl aldehydes, dimedone, ethyl acetoacetate, ammonium acetate	Solvent-free, 75 °C, 3 mg catalyst	6–22 min	81%–96%	Biomass-derived, magnetically recoverable nanocatalyst; very low catalyst loading
21	Green (Catalyst-free) Patil et al. (2017)	No catalyst	Dimedone, ammonium acetate, aromatic aldehyde, malononitrile	Water only	–	–	Fully external catalyst-free green synthesis
22	Green (Catalyst-Free) Devi et al. (2020)	Ultrasonication assisted synthesis	Dimedone, benzaldehydes, benzyl acetoacetate, and ammonium acetate	Ethanol, ultrasonic irradiation at room temperature	10 min	92.98%	One-pot multicomponent,non-catalyzed reaction

### 2.3 Structural dominance of 5-oxo HHQs

Most reported synthetic routes for hexahydroquinolines consistently yield 5-oxo derivatives, and this dominance is largely driven by the chemistry of the starting materials and the mechanism of the Hantzsch-type condensation. Most methods employ 1,3-cyclohexanediones, especially dimedone, which already contain a carbonyl at the position that becomes C-5 in the final scaffold (Ranjbar and Khonkarn, 2019; Rostamnia et al., 2013; Çetin et al., 2022). During the multicomponent reaction, this carbonyl is preserved and also stabilizes key intermediates such as enaminones and Michael adducts, making the 5-oxo pathway both kinetically accessible and thermodynamically favored. Even when alternative conditions are used, the system tends to tautomerize toward the keto form at C-5, reinforcing its stability (Patil et al., 2017). As a result, the prevalence of 5-oxo-HHQs stems from their synthetic accessibility and the tendency of reaction pathways to favor this core structure under varied conditions.

### 2.4 Alternative synthetic routes for underexplored HHQ chemotypes

#### 2.4.1 2-Oxo hexahydroquinolines

##### 2.4.1.1 Enamine-mediated synthesis of 2-oxo HHQ derivatives

Enamine-mediated strategies have emerged as versatile approaches for constructing HHQs derivatives, leveraging the high nucleophilicity of the enamine α-carbon to facilitate acylation and cyclization.

**SCHEME 20:**
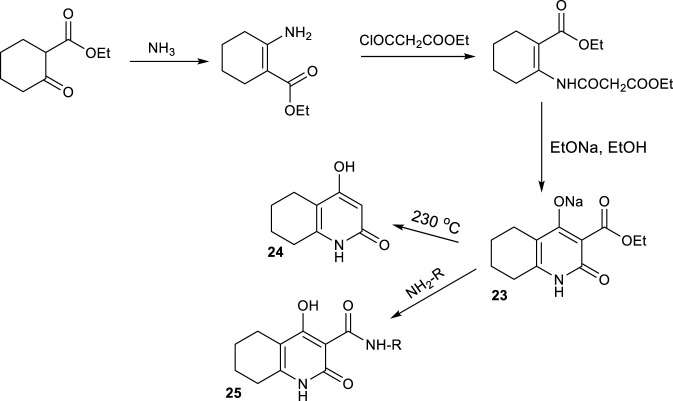
Enamine mediated synthesis of 2-oxo HHQs with ammonia.

Ukrainets et al. (2006) reported the synthesis of 4-hydroxy-2-oxo-1,2,5,6,7,8-hexahydroquinoline-3-carboxylic acid esters through a modular and efficient approach. The synthesis began by reacting ethyl cyclohexanone-2-carboxylate with ammonia to form the enamine intermediate (Scheme 20). This was subjected to condensation with the ethyl ester of chlorocarbonylacetic acid to yield a diester intermediate, which underwent intramolecular cyclization under basic conditions to generate the HHQ core. Subsequent amidation with various hetarylamines produced a series of structurally diverse 4-hydroxy-2-oxo-hexahydroquinoline hetarylamides.

**SCHEME 21:**
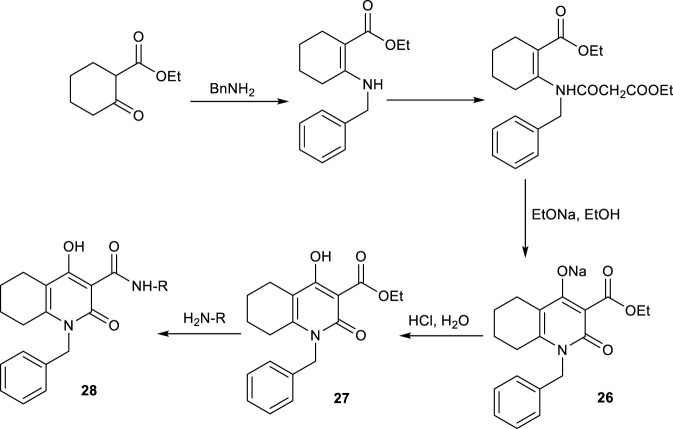
Enamine mediated synthesis of 2-oxo HHQs with benzylamine.

Similarly, Davidenko (2015) synthesized 1-benzyl-4-hydroxy-2-oxo-1,2,5,6,7,8-hexahydroquinoline-3-carboxamides using the same ethyl cyclohexanone-2-carboxylate which was condensed with benzylamine to form an enamine intermediate (Scheme 21). This was followed by acylation and intramolecular cyclization to afford the hexahydroquinoline ester (2-oxo HHQ scaffold), which was subsequently converted to a series of 3-carboxamide derivatives via thermolysis with various anilines or heteroarylamines in dimethylformamide. The reactions yield crystalline 1-benzyl-4-hydroxy-2-oxo-hexahydroquinoline-3-carboxamides as the final products.

##### 2.4.1.2 Ultrasound assisted synthesis of 2-oxo HHQ derivatives

**SCHEME 22:**
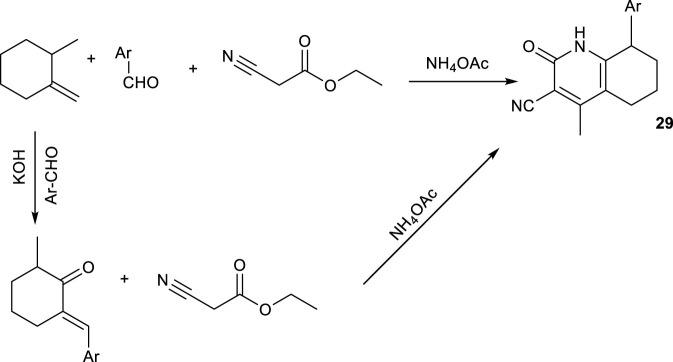
Ultrasound assisted synthesis of 2-oxo HHQs.

Another study by Faidallah and Al-Juaid (2014) employed ultrasound irradiation in boiling ethanol to facilitate the one-pot synthesis of 3-cyano-8-methyl-2-oxo-4-substituted hexahydroquinolines. The method involved reacting the corresponding aldehyde, 2-methylcyclohexanone, ammonium acetate, and ethyl cyanoacetate, with the mixture sonicated in a water bath for 15 min (Scheme 22). The use of 2-methylcyclohexanone directs the oxo functionality to the C-2 position during cyclization, while the ultrasound-assisted conditions accelerate the reaction and enhance product yield. The overall reaction time was reduced to 6–8 h, shorter than the compared method (which was a two-step reaction) and produced yields ranging from 78% to 92%.

##### 2.4.1.3 Synthesis of functionalized 2-oxo HHQs with tetracyanoethylene

**SCHEME 23:**
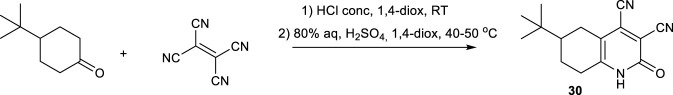
Synthesis of 2-oxo HHQs with tetracyanoethylene.

Fedoseev et al. (2022) synthesized 2-oxo-6-tert-butyl-1,2,5,6,7,8-hexahydroquinoline-3,4-dicarbonitrile via a one-pot reaction of cyclohexanone with tetracyanoethylene (Scheme 23). The reaction forms a tetracarbonitrile intermediate, which cyclizes under acidic aqueous conditions through terminal cyano groups, leaving C4 cyanos intact. Subsequent dehydration and partial aromatization generate the 2-oxo hexahydroquinoline core. This one-pot method only simplified the procedure and improved efficiency, yielding 85% of the HHQ derivative, compared to 76% in the stepwise approach.

On the other hand, rather than the *de novo* synthesis of the 2-oxo HHQ core, Smirnova et al. (1999) and Albov et al. (2004) utilized pre-formed 2-oxo hexahydroquinolines as scaffolds (2-oxo-1,2,5,6,7,8-hexahydroquinoline-3-carbonitrile and 2-oxo-1,2,5,6,7,8-hexahydroquinoline-4-carboxylic acid respectively), focusing on their functionalization and cyclization for the synthesis of HHQ derivatives.

#### 2.4.2 1,2,3,4, 4a, 5- hexahydroqunolines

**SCHEME 24:**
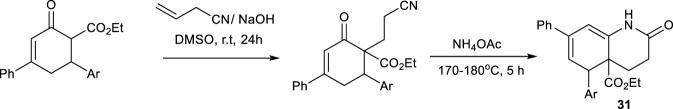
Synthesis of 1,2,3,4,4a,5-hexahydroquinoline.

Popandova-Yambolieva (1990) developed a two-step route to synthesize substituted 1,2,3,4,4a,5-hexahydroquinolin-2-ones. In their approach, ethyl 6-aryl-4-phenyl-2-oxo-3-cyclohexene-1-carboxylates were first subjected to a mild Michael addition with acrylonitrile in the presence of aqueous NaOH, to give β-cyano intermediates, which were then cyclized with ammonium acetate to afford the corresponding 2-oxo-hexahydroquinolinones (Scheme 24). This method is distinctive because it provides access to the less common 1,2,3,4,4a,5-hexahydroquinoline reduction pattern, offering structural motifs that are not typically obtained through classical Hantzsch-type syntheses.

The key synthetic strategies for hexahydroquinoline chemotypes that are less frequently reported in the literature are summarized in Table 2.

**TABLE 2:** Table summarizing the synthesis of the underexplored hexahydroquinolines chemotypes.

S/N	Category	Key reagents	Time	Yield	Unique advantage
1	Enamine-Mediated (Davidenko, 2015)	Ethyl cyclohexanone-2-carboxylate, benzylamine, anilines/heteroarylamines	13–15 h	85%–95%	Modular approach; allows structural diversity; crystalline 3-carboxamide derivatives
2	Enamine-Mediated (Ukrainets et al., 2006)	Ethyl cyclohexanone-2-carboxylate, ammonia, ethyl ester of chlorocarbonylacetic acid, hetarylamines	13–15 h	76%–93%	Multistep approach, produces structurally diverse 4-hydroxy-2-oxo HHQ hetarylamides
3	Ultrasound-Assisted (Faidallah and Al-Juaid, 2014)	Aldehyde, 2-methylcyclohexanone, ammonium acetate, ethyl cyanoacetate	6–8 h	78%–92%	One-pot, faster reaction, oxo group directed to C-2, enhanced yields
4	Tetracyanoethylene-Mediated (Fedoseev et al., 2022)	Cyclohexanone, tetracyanoethylene	1.5–2 h	85%	Simplified procedure; improved efficiency compared to stepwise method
5	Functionalization of Pre-formed 2-oxo HHQs (Albov et al., 2004; Smirnova et al., 1999)	2-oxo-1,2,5,6,7,8-hexahydroquinoline-3-carbonitrile/2-oxo-1,2,5,6,7,8-hexahydroquinoline-4-carboxylic acid	–	–	Enables derivatization without full *de novo* synthesis
6	Two-step 1,2,3,4,4a,5-HHQ synthesis (Popandova-Yambolieva, 1990)	Ethyl 6-aryl-4-phenyl-2-oxo-3-cyclohexene-1-carboxylate, acrylonitrile, ammonium acetate	5–6 h	21%	Access to less common 1,2,3,4,4a,5-HHQ scaffolds not obtained by classical Hantzsch methods

## 3 Pharmacological activities of HHQ derivatives

HHQ derivatives have attracted significant attention due to their broad spectrum of biological activities. The scaffold is promising in medicinal chemistry because its partially saturated quinoline core supports diverse chemical substitutions without disrupting the stability of the ring system (Ranjbar and Khonkarn, 2019). This structural flexibility allows HHQ derivatives to influence several biological pathways, including oxidative stress responses, enzyme activity, ion-channel function, and receptor-mediated signaling (Mirani Nezhad et al., 2022; Zarghi et al., 2014; Şimşek et al., 2008). This section summarizes the major reported biological and pharmacological effects of HHQs.

### 3.1 Antiprotozoal activities of hexahydroquinolines

Protozoal infections remain a major global health challenge, particularly in resource-limited tropical and subtropical regions where diseases such as malaria, leishmaniasis, toxoplasmosis, and trypanosomiasis continue to cause significant morbidity and mortality (Cosma et al., 2024; Khan et al., 2018; Rizwan et al., 2021; Vanaerschot et al., 2017). Current therapeutic options are severely constrained by multiple factors including a limited arsenal of effective drugs, increasing drug resistance (particularly concerning in malaria and leishmaniasis), prohibitive treatment costs, severe adverse effects, and lengthy treatment regimens that compromise patient compliance (Kaushik et al., 2025; Rizwan et al., 2021). These challenges have intensified interest in small, drug-like scaffolds capable of modulating diverse parasite targets while maintaining favorable pharmacokinetic profiles (Girault et al., 2000; Lee et al., 2019; Wu et al., 2021). Recent studies have reported HHQ derivatives with promising activity against a range of protozoa, highlighting their potential as multi-target antiprotozoal scaffolds (Molaei et al., 2023; Oduselu et al., 2024; Zahedi et al., 2020). This section streamlines the focus to the major reported antiprotozoal activities of HHQs.

#### 3.1.1 Malaria

Malaria, caused by *Plasmodium* parasites and transmitted by female Anopheles mosquitoes, is a major global health challenge, particularly in sub-Saharan Africa (Diagana and Jones, 2017; Oduselu et al., 2024). Current antimalarial therapies primarily target the symptomatic blood-stage parasites to clear the infection, this allows gametocytes at the sexual stage to remain infectious to mosquitoes, contributing to widespread transmission and the emergence of drug-resistant strains. Preventing human-to-mosquito transmission is therefore critical for effective malaria control and eventual eradication (Oduselu et al., 2024; Vanaerschot et al., 2017). HHQs have emerged as a promising class of antiplasmodial compounds, exhibiting potent activity against both asexual blood stages of drug-sensitive and multidrug-resistant parasites with minimal mammalian cytotoxicity. Importantly, HHQs also block the development of male gametocytes and gametes, effectively preventing parasite transmission. This dual curative and transmission-blocking activity positions HHQs as high-value candidates for combination therapies, offering a strategy to simultaneously treat infection and curb malaria spread (Oduselu et al., 2024; Vanaerschot et al., 2017; Veale and Müller, 2020).

**FIGURE 2:**
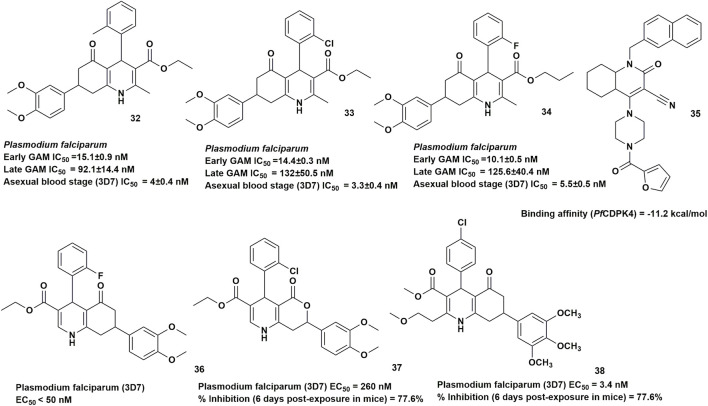
HHQ derivatives with antimalarial activity.

Vanaerschot et al. (2017) were the first to report HHQs as a novel class of dual-acting antimalarial compounds through phenotypic screening of 3,825 compounds from the Novartis–GNF Malaria Box against early- and late-stage *Plasmodium falciparum* gametocytes using luciferase-based and high-content imaging assays, respectively. Four lead gametocytocidal compounds were obtained, three of which shared a common hexahydroquinoline scaffold (**compounds 32–34**) and exhibited IC_50_ values <200 nM against both early and late gametocyte stages (Figure 2). These HHQs, denoted as GNF-Pf-5640, GNF-Pf-5660, and GNF-Pf-5668 respectively, also showed potent asexual blood-stage activity, with IC_50_ values <25 nM against drug-sensitive 3D7 and multidrug-resistant Dd2 parasites, including a PfATP4 mutant line. Dual gamete formation assays demonstrated strong activity against mature male gametocytes and developing male gametes, with marked inhibition of exflagellation following 24 h exposure, while no activity was observed on female gamete formation; nevertheless, this male-specific activity translated into effective transmission blocking, as reflected by reduced oocyst prevalence and intensity in *Anopheles* mosquitoes. Mechanistic studies indicated that the HHQs disrupt hemoglobin metabolism by inhibiting host hemoglobin uptake and reducing hemozoin and total heme levels within intra-erythrocytic parasites. *In vivo*, they suppressed asexual blood-stage growth in *Plasmodium berghei*–infected mice in a 4-day Peters test at 100 mg/kg. Cytotoxicity assays showed no detectable inhibition at 40 μM for compound **4** whereas the other two HHQs induced approximately 50% cytotoxicity at the same concentration.

In another study by Oduselu et al. (2024), a similarity search on PubChem identified 16,525 hexahydroquinolines, which were filtered using structure-based virtual screening for inhibitory activity against *Pf*CDPK4, an essential target involved in sexual stage development, more specifically the exflagellation of male gametocytes in mosquitoes. Ten HHQs were reported to exhibit better binding affinity compared to various reference compounds implying prospective human-mosquito malaria transmission-blocking potential. The top-performing molecule, Compound **35** (Figure 2), demonstrated the strongest predicted binding affinity (−11.2 kcal/mol, compared with −6.8 to −9.9 kcal/mol for the reference compounds), maintained structural stability during a 200 ns molecular dynamics simulation, and displayed favorable predicted ADMET properties.

Also in a recent lead optimization study based on high-throughput screening at St. Jude Children’s Research Hospital, Compound **36** (Figure 2) was identified as a top compound that inhibited male *in vitro* Plasmodium gamete formation and the reduction of oocyst infection and prevalence in Anopheles mosquitoes (Horn et al., 2024). Based on this lead compound scaffold, an extensive structure–activity relationship (SAR) analysis was conducted, involving modifications at the 2-, 3-, 4-, 6-, and 7-positions of the lead core. At the end of the study, compound **37** and **38** demonstrated the most promising efficacy, reducing parasitemia by more than 75% by day 6.

#### 3.1.2 Leishmaniasis

Leishmaniasis is a neglected tropical disease caused by over twenty species of protozoan parasites of the genus *Leishmania*, transmitted to humans through the bite of infected female phlebotomine sandflies (Mann et al., 2021; Pareyn et al., 2025). It affects millions worldwide, with around one billion people at risk (Majoor et al., 2025; Makarani et al., 2025), and can present as cutaneous (CL), mucocutaneous (MCL), or visceral leishmaniasis (VL), depending on the species and host factors (Cosma et al., 2024; Makarani et al., 2025; Pareyn et al., 2025). In the Americas, *L. amazonensis* is a major cause of CL, while *L. major* predominates in the Old World, including Asia, Africa, and the Middle East (Cosma et al., 2024; Mann et al., 2021). The disease is often difficult to diagnose early due to slow progression and extensive skin involvement in CL (Mann et al., 2021). Available treatments, including pentavalent antimonials, amphotericin B, miltefosine, and paromomycin, are limited by toxicity, high cost, and emerging drug resistance (Majoor et al., 2025; Makarani et al., 2025). Given these, there has been growing interest in exploring novel chemical scaffolds for antileishmanial activity.

**FIGURE 3:**
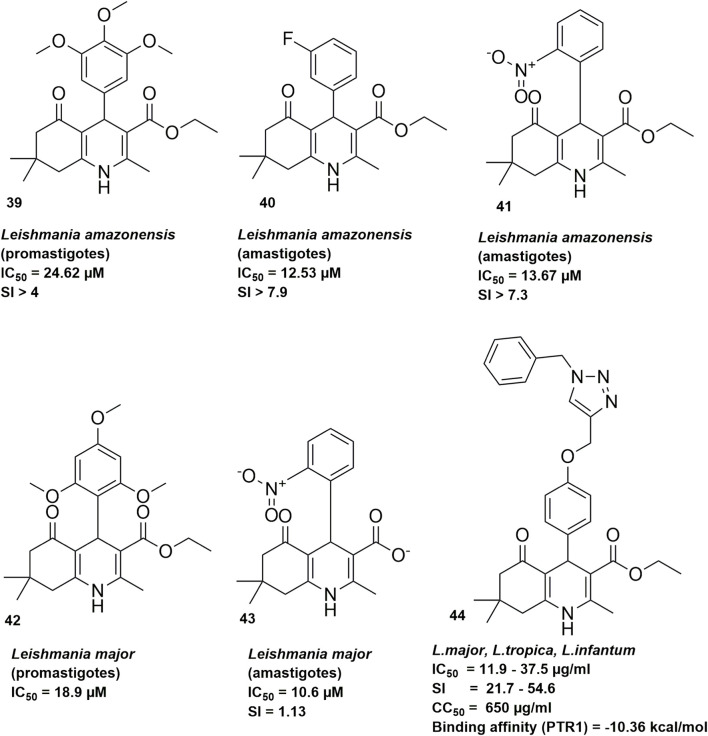
HHQ derivatives with dual-stage antileishmanial activity.

Oliveira et al. (2024) synthesized a series of twenty-three 1,4-dihydropyridine (1,4-DHP) derivatives and evaluated their activity against *Leishmania amazonensis* promastigotes. Of these, eleven compounds inhibited parasite motility by more than 60%, with eight showing IC_50_ values below 50 µM. The most potent compound against promastigotes was Compound **39** (Figure 3), an HHQ bearing methoxy groups on the aromatic ring, which exhibited an IC_50_ of 24.62 µM and a Selectivity Index (SI) greater than 4, indicating favorable activity with limited toxicity toward mammalian cells. HHQ compounds **40** and **41** (Figure 3) were the most effective against the intracellular amastigote stage, with IC_50_ values of 12.53 µM and 13.67 µM, respectively, and high SI (>7). SAR analysis showed that methoxy substitutions on the aromatic ring could enhance antileishmanial activity, but their presence alone was not sufficient to ensure efficacy. Halogenation conferred moderate activity, as observed for compound **40**, while the position of nitro groups influenced potency, exemplified by compound **41** The antileishmanial activity of the nitroaromatic HHQs may be related to their redox properties and ability to generate reactive oxygen species, disrupting mitochondrial potential within the parasite.

Later in another study, Oliveira and coworkers (2025) investigated the *in vitro* activity of another twenty-four synthesized 1,4-dihydropyridine (1,4-DHP) against *Leishmania major* promastigotes and amastigotes. Initial screening against extracellular promastigotes identified five active compounds, with compound **42** (Figure 3) showed the lowest IC_50_ of 18.9 μM. Testing against intracellular amastigotes revealed that also five compounds inhibited parasite growth at IC_50_ values below 50 μM, with compound **43** (Figure 3) (IC_50_ of 10.6 μM) displaying the highest selectivity for amastigotes over macrophages (SI = 1.13). Despite being the most selective, this value is still low especially compared to amphotericin B standard (SI = 17.2), this indicates a narrow therapeutic window and the need for further optimization to improve safety and efficacy. Structure–activity relationship analysis indicated the presence of fluorine, nitro, methoxy, and aromatic ether groups enhanced antiparasitic effects, while symmetry of the 1,4-DHP core in the HHQ often reduced potency.

Molaei et al. (2023) also reported the synthesis of ten novel hexahydroquinoline-1,2,3-triazole hybrid compounds and their antileishmanial activity against amastigote and promastigote forms of *L. major*, *L. tropica*, and 2 *L. infantum* strains. Among the derivatives, compound **44** (Figure 3) exhibited the most potent activity with IC_50_ values ranging from 11.9 to 37.5 μg/mL against both promastigote and amastigote forms and lowest cytotoxicity, significantly outperforming the standard drugs amphotericin B and Glucantime. Cytotoxicity assessment on J774.A1 macrophages revealed favorable safety profiles, with compound **15** displaying a CC_50_ of 650 μg/mL and exceptional selectivity indices (21.7–54.6), considerably higher than Glucantime (10.4–14.4). Structure-activity relationship analysis indicated that the presence of two methyl groups at position 7 of the quinoline ring and an unsubstituted benzyl moiety on the triazole were crucial for enhanced activity, while electron-withdrawing substituents diminished potency. To elucidate the molecular mechanism underlying the observed antileishmanial activity, the authors conducted molecular docking and molecular dynamics simulation studies (100 ns) targeting pteridine reductase 1 (PTR1), a validated drug target in *Leishmania* parasites. Compound **44** exhibited the highest docking score with a binding energy of −10.36 kcal/mol and formed five crucial hydrogen bonds with Gly225, Phe113, and the NADPH cofactor as well as stable binding throughout the simulation (RMSD ∼0.25–0.35 nm).

#### 3.1.3 Toxoplasmosis

Toxoplasmosis is a widespread apicomplexan parasitic infection caused by *Toxoplasma gondii*, affecting roughly one-third of the global population (Khairullah et al., 2024). Transmission occurs mainly through ingestion of oocysts from contaminated food, water, or soil, and via tissue cysts in undercooked meat (Farhab et al., 2025; Wu et al., 2021). While most infections are asymptomatic, severe disease can occur in immunocompromised individuals and during congenital infection, leading to neurological or ocular complications (Farhab et al., 2025; Khairullah et al., 2024). The parasite’s ability to form chronic tissue cysts in muscle and brain makes eradication difficult (Khairullah et al., 2024), creating a continued need for new compounds with potent activity against both acute and latent stages of *T. gondii* especially in the face of growing resistance (Liu et al., 2024; Wu et al., 2021).

**FIGURE 4:**
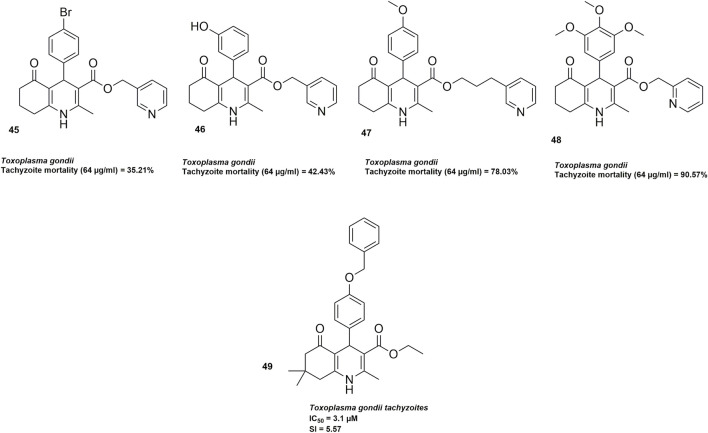
HHQs with demonstrated activity against tachyzoites of *Toxoplasma gondii*.

Zahedi et al. (2020) synthesized four 5-oxo-hexahydroquinoline derivatives and evaluated their anti-*Toxoplasma gondii* activity through *in vitro*, *in vivo*, and molecular docking studies. *In vitro* assays using flow cytometry showed that **47** and **48** (Figure 4) were the most potent, causing up to 78% and 90% tachyzoite mortality at 64 μg/mL, respectively, while **45** and **46** (Figure 4) displayed moderate activity (∼42% maximum mortality). *In vivo* studies in BALB/c mice confirmed dose-dependent efficacy, with **47** slightly outperforming **48** in extending survival without observed toxicity. Compound **47** extended survival up to 8.6 days and **48** up to 9.6 days at the highest dose, without observable toxicity, compared to 5.4 days in untreated controls. Molecular docking studies targeting the enoyl-acyl carrier protein reductase (ENR) enzyme, a key component of the apicoplast FAS-II pathway, supported these findings. Compound **48** exhibited the most favorable binding energy at −9.2 kcal/mol, slightly better than the reference ligand triclosan (−9.1 kcal/mol), while **47** scored −8.5 kcal/mol. Taken together, compounds **47** and **48** showed strong potential as anti-*T. gondii* leads, warranting further optimization and pharmacokinetic studies to enhance their efficacy.

Oliveira et al. (2025) further conducted antitoxoplasmosis activity studies against the 24 synthesized 1,4 DHPs. *Toxoplasma gondii* tachyzoites (RH strain) were cultured in Vero cells, and the compounds were screened for inhibitory activity, revealing that 11 compounds exhibited EC_50_ values below 50 μM, with compound **49** (an HHQ) showing the highest selectivity index (SI = 5.57) at an EC_50_ of 3.1 μM. To explore the molecular basis of this activity, **49** (Figure 4) was docked against *T. gondii* calcium-dependent protein kinase-1 (TgCDPK1), it had a binding affinity of −7.174 kcal/mol. Further *in silico* ADME-T and drug-likeness assessments indicated that **49** had high predicted intestinal absorption (HIA = 96.17%), favorable predicted Caco-2 permeability, moderate blood–brain barrier penetration and no predicted mutagenicity, although potential hepatotoxicity was flagged.

#### 3.1.4 Chagas disease

**FIGURE 5:**
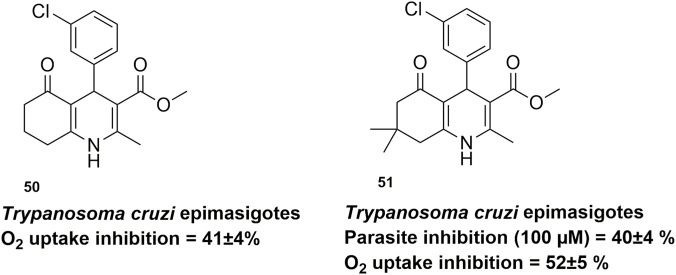
HHQs with moderate activity against *Trypanosoma cruzi* epimastigotes in Chagas disease.

In 2000, Maya and colleagues investigated the trypanosomicidal activity of 3-chloro-phenyl-1,4-dihydropyridine derivatives, including hexahydroquinolines (compounds **50** and **51**), against Trypanosoma cruzi epimastigotes of the Brener, Tulahuen, and LQ strains (Maya et al., 2000). However, hexahydroquinolines with a fused-ring system exhibited markedly lower antiparasitic activity than non-fused 1,4-dihydropyridines. At a concentration of 10 μM, nicardipine (positive control) inhibited parasite growth by approximately 40%, whereas hexahydroquinoline **51** (Figure 5) produced only about 10% inhibition, and compound **52** showed no detectable activity. At 100 μM, nicardipine achieved nearly complete growth inhibition (98%), while compound **51** showed only moderate improvement (40%), and compound **52** remained inactive. Despite their limited effect on parasite growth, both hexahydroquinolines significantly inhibited oxygen uptake by 41% and 52%, respectively. The authors concluded that fused-ring hexahydroquinolines possess only moderate trypanosomicidal activity, with ring fusion negatively affecting potency compared to simpler 1,4-dihydropyridine analogues.

### 3.2 Anticancer activity

Ranjbar and Khonkarn (2019) synthesized twelve novel 5-oxo-hexahydroquinoline derivatives to tackle multidrug resistance (MDR) in cancer cells driven by the overexpression of ATP-binding cassette (ABC) transporters which lower intracellular drug concentrations. These compounds were examined for their ability to reverse MDR using flow cytometry, doxorubicin resistance assays, cell viability tests, and glutathione content measurements.

**FIGURE 6:**
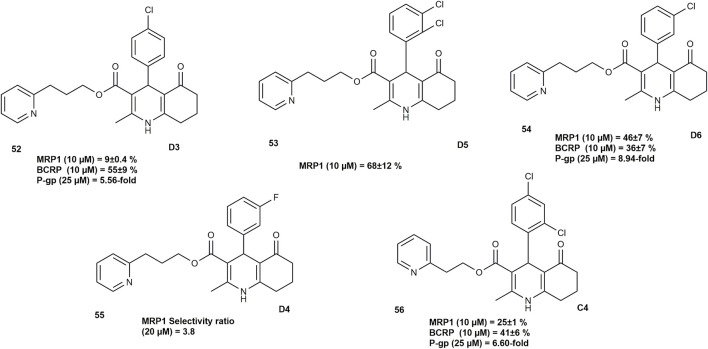
HHQ with multi-drug resistance (MDR) reversal potential in cancer.

All compounds met Lipinski’s and Veber’s criteria for oral drug-likeness and several derivatives exhibited strong efflux pump inhibitory activity, with distinct substituent-dependent selectivity. Compounds bearing chloro-substituted phenyl groups at the C4 position showed the most pronounced inhibitory effects: **D6, D5, and D3** (Figure 6) best inhibited P-glycoprotein (P-gp), multidrug resistance-associated protein 1 (MRP1), and breast cancer resistance protein (BCRP), respectively at low micromolar concentrations of 1–10 μM (for MRP1 and BCRP) and 25 μM (for P-gp). In contrast, the 3-fluorophenyl analogue **D4** (Figure 6) was the most potent inducer of MRP1-dependent collateral sensitivity. Overall, the chlorine-containing derivatives **(D6, C4, and D3)** exhibited broad inhibitory activity across all three ABC transporters, with D6 additionally triggering collateral sensitivity via reduced glutathione efflux.

**FIGURE 7:**
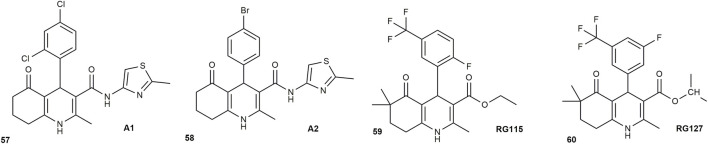
HHQ derivatives with anticancer activity.

In a related study carried out by Shahraki et al. (2020), a new series of sixteen 1,4,5,6,7,8-hexahydro-5-oxoquinoline-3-carboxamide derivatives (A series), incorporating a 4-methylthiazole moiety, and their corresponding tetrahydroquinoline analogues (B series) were synthesized with the goal of assessing their potential to counteract multidrug resistance (MDR) in cancer cells. Compounds **A1** and **A2** (Figure 7) with 2,4-dichlorophenyl and 4-bromophenyl substituents respectively exhibited the strongest P-glycoprotein (P-gp) inhibitory activity. Nevertheless, oxidation of the hexahydroquinoline ring of these compounds to the tetrahydroquinoline structure in the B series did not significantly affect MDR reversal. However, selective toxicity toward cancer and multidrug-resistant cells, relative to non-resistant and non-cancerous cells, was observed only for the 4-bromophenyl derivatives A2.

Çetin et al. (2022) also synthesized two novel hexahydroquinoline (HHQ) derivatives and their magnetic nanoparticle (MNP) conjugates to evaluate their potential as anticancer lead compounds. The study further examined how conjugating these compounds to magnetic nanoparticles affected their activity. Silica-coated magnetic nanoparticles (Fe_3_O_4_@SiO_2_) were prepared using the co-precipitation method, followed by coating with silica. These nanoparticles were then functionalized with an epoxy-silane agent (GPTS) and linked to the HHQ compounds, named **RG115** and **RG127** (Figure 7). The anticancer activities of both the free compounds and their MNP conjugates were evaluated against the MCF-7 breast cancer cell line at concentrations of 1, 5, and 10 mg/mL. **RG115** alone showed no cytotoxicity at any of the tested doses. However, when conjugated to magnetic nanoparticles (RG115MNP), it exhibited strong anticancer activity, with the highest dose (10 mg/mL) showing cytotoxic effects, and lower doses (1 and 5 mg/mL) showing anticancer activity. **RG127** in both free and nanoparticle-conjugated forms (RG127MNP), demonstrated anticancer effects at all concentrations tested. Notably, at 10 mg/mL, RG115MNP showed the strongest effect, with 91.5% cancer cell inhibition, compared to 78.4% for **RG127**% and 79.9% for RG127MNP. The study ultimately showed that attaching hexahydroquinoline derivatives to magnetic nanoparticles significantly enhanced their anticancer activity.

### 3.3 Antimicrobial activity

**FIGURE 8:**
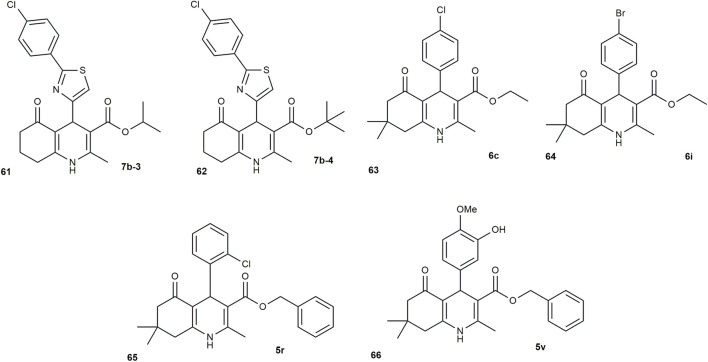
HHQ derivatives with antibacterial and antifungal activity.

HHQs exhibit broad-spectrum antimicrobial activity, acting against diverse bacterial and fungal pathogens. Their activity is strongly influenced by substituents on the HHQ scaffold, which modulate key biological interactions and these features make HHQs credible starting points for new anti-infective agents (Ranjbar and Khonkarn, 2019). In 2008, Lak et al. reported the ability of 3-alkyl and 3-aryl ester derivatives of HHQs to reduce bacterial resistance to ciprofloxacin. The results showed that two 3-alkyl esters, compounds **7b-3** and **7b-4** (Figure 8), enhanced the antibacterial effect of ciprofloxacin. Among them, compound **7b-4** had a stronger effect, increasing the ciprofloxacin inhibition zone by 5.61-fold on agar plates. However, none of the 12 synthesized compounds showed direct antibacterial activity on their own at the tested concentration. A similar study by Pourmand et al. (2014) investigated how hexahydroquinoline derivatives affect the activity of ciprofloxacin and the expression of the norA efflux pump gene in methicillin- and ciprofloxacin-resistant *Staphylococcus aureus* (MCRSA). The findings showed that when combined with the derivative, the minimum inhibitory concentration (MIC) of ciprofloxacin decreased by fourfold and twofold when used with 1/2 MIC and 1/4 MIC of the derivative, respectively. These results suggest that hexahydroquinoline derivatives may have potential as helper compounds in combination therapies to improve the effectiveness of existing antibiotics.

Morbale et al. (2015) used the agar well diffusion method to investigate the *in vitro* antimicrobial activity of hexahydroquinolines derivatives (6a–6j) against *Staphylococcus aureus*, *Escherichia coli*, *Aspergillus flavus*, and *Candida albicans*, with ciprofloxacin and fluconazole as reference drugs. Among these, the para-bromo **(6i)** derivative showed strong antibacterial and antifungal activity, while para-chloro **(6c)** derivative demonstrated potent effects, particularly against *S. aureus* and *A. flavus* (Figure 8). Derivatives 6b (ortho-chloro) and 6f (ortho-nitro) retained moderate activity. Mirani Nezhad et al. (2022) also explored the *in vitro* antibacterial activity of several hexahydroquinoline derivatives (5c, 5i, 5j, 5r, 5s, 5o, and 5v) against *Escherichia coli* and *Bacillus subtilis*. Among the tested compounds, **5r** and **5v** (Figure 8) demonstrated notable antibacterial effects against both strains, while compounds 5i and 5o showed no activity. The remaining derivatives displayed moderate or limited activity.

**FIGURE 9:**
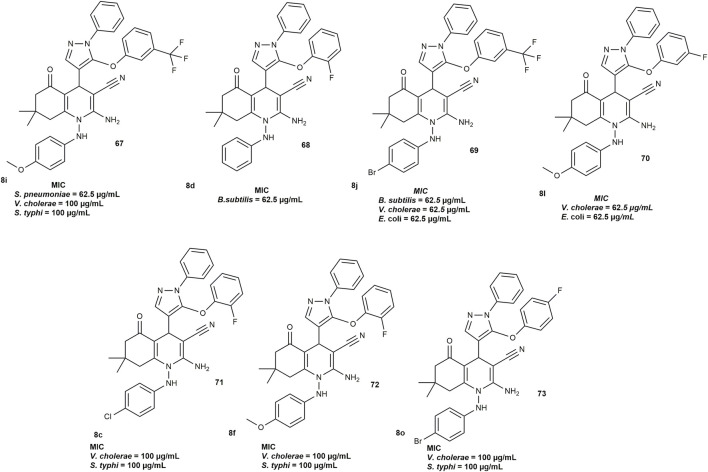
Fluorinated HHQs with antibacterial activity.

Karad et al. (2015) also assessed the antimicrobial activity of fluorinated hexahydroquinoline derivatives using the broth microdilution method. The compounds were tested against six bacterial strains: three Gram-positive (*Bacillus subtilis*, *Clostridium tetani*, *Streptococcus pneumoniae*) and three Gram-negative (*Salmonella typhi*, *Escherichia coli*, *Vibrio cholerae*), as well as two fungal strains (*Aspergillus fumigatus*, *Candida albicans*). Standard drugs, including ampicillin, norfloxacin, ciprofloxacin, and griseofulvin were used for comparison. Several compounds showed strong antibacterial activity. Compound **8i** (Figure 9) was more effective against *S. pneumoniae* with a MIC of 62.5 μg/mL compared to ampicillin at 100 μg/mL. Compounds **8d** and **8j** were most active against *B. subtilis* with MIC values of 62.5 μg/mL, better than both ampicillin and norfloxacin. For Gram-negative bacteria, compounds **8j** and **8L** showed higher potency against *E. coli* and *V. cholerae* than ampicillin (MIC = 100 μg/mL). Compounds **8c, 8f, 8i,** and **8o** (Figure 9) demonstrated activity comparable to ampicillin against *S. typhi* and *V. cholera* at the same concentration.

### 3.4 Cardiovascular activity (calcium channel modulatory activity)

**FIGURE 10:**
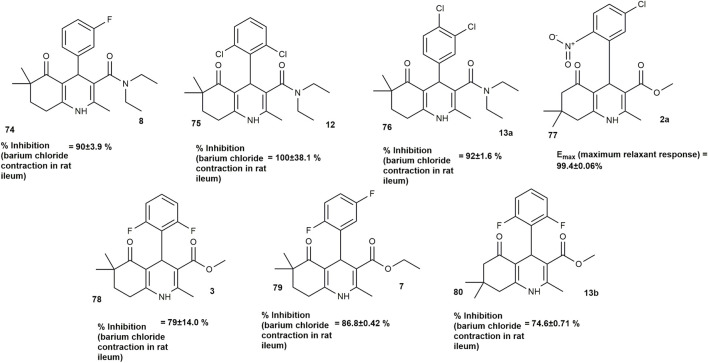
HHQ derivatives with calcium channel blocking potential.

HHQs share certain structural and electronic features with 1,4-dihydropyridines, despite having a fused bicyclic scaffold. These similarities support the possibility of calcium-channel interactions within the HHQ class (Şimşek et al., 2008). A series of thirteen 2,6,6-trimethyl-3-carbamoyl-4-aryl-5-oxo-1,4,5,6,7,8-hexahydroquinoline derivatives were synthesized via the Hantzsch reaction by Kismetli et al. (2004) and evaluated for their calcium channel blocking potential. Pharmacological assessment using isolated rat ileum preparations revealed that, at a concentration of 10^−5^ mol/L, compounds **8, 12**, and **13a** (Figure 10) demonstrated greater activity than the reference drug nicardipine. At 10^−4^ mol/L, compound **12** surpassed the activity of nicardipine, while compounds **8** and **13a** exhibited comparable potency. Structure–activity relationship (SAR) analysis showed that both the nature and the position of substituents on the aryl ring played a critical role in modulating calcium channel blocking efficacy.

Şimşek et al. (2008) also synthesized twelve hexahydroquinoline derivatives to investigate the effect of two different electron-withdrawing groups on calcium channel modulation. The most active compound, **2a** (Figure 10), showed the same maximum relaxant effect as nifedipine but was less potent.

In a related study, Gupta and Misra (2008) synthesized 34 HHQ derivatives and showed that most of the compounds showed more potent calcium antagonistic activity than nicardipine on isolated rat ileum precontracted with barium chloride. Specifically, compounds **3, 7**, and **13b** (Figure 10) demonstrated higher potency at a concentration of 10^−5^ mol/L, with compound **7** showing the highest inhibition (86.8%) compared to nicardipine (69.6%)

### 3.5 Anti-inflammatory activity

**FIGURE 11:**
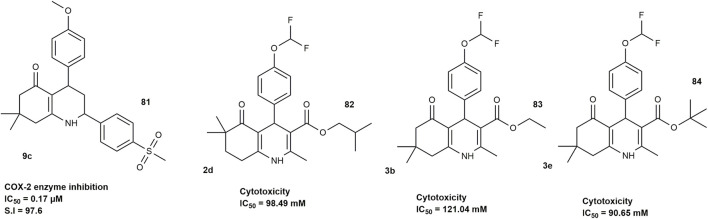
HHQ derivatives with anti-inflammatory activity.

A study by Zarghi et al. (2014) investigated 5-oxo-1,4,5,6,7,8-hexahydroquinoline derivatives for their ability to selectively inhibit cyclooxygenase-2 (COX-2), an enzyme linked to inflammation The results showed that both the position of the SO_2_Me group (a known COX-2-targeting feature) and the type of other substituents played an important role in determining how well the compounds worked and how selective they were for COX-2. Compound **9c** (Figure 11) was seen as the most promising, with strong COX-2 inhibition (IC_50_ = 0.17 µM) and a high selectivity index (S.I. = 97.6), which was close to that of the standard drug celecoxib (IC_50_ = 0.05 µM; S.I. = 405).

Pehli̇Vanlar et al. (2024) similarly investigated the cytotoxicity, intracellular ROS production, and inhibitory activity against inflammatory mediators and complement proteins of hexahydroquinolines. They synthesized fifteen hexahydroquinoline-3-carboxate derivatives using the Hantzsch method. The MTT assay results showed that compounds **2d, 3b** and **3e** (Figure 11) were the least cytotoxic among the fifteen tested in NIH 3T3 mouse fibroblasts. It was reported that these compounds did not significantly reduce IL-1α, IL-10, or TNF-α levels in LPS-induced cells but did cause a marked decrease in TGF-β1 levels, with compound 3e showing the strongest effect. Additionally, compound **3e** was found to slightly increase C3 levels (not significant) and significantly boost complement C9 levels, suggesting a possible benefit in membrane attack complex (MAC) formation. Compounds **2d** and **3b** reportedly led to minor, non-significant decreases in both C3 and C9 levels.

### 3.6 Antioxidant activity

From the HHQ compounds synthesized by Mirani Nezhad et al. (2022), 11 derivatives exhibited significant antioxidant activity. They tested the derivatives using the DPPH radical scavenging assay and results revealed scavenging efficiencies ranging from 75% to 98%.

### 3.7 Antitubercular activity

**FIGURE 12:**
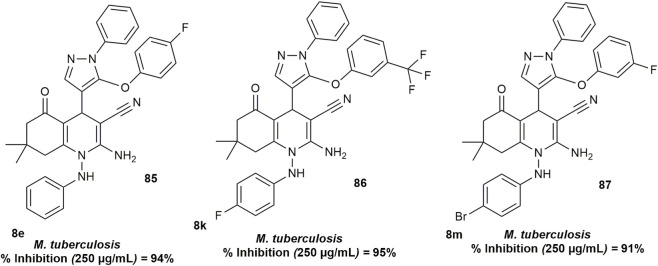
HHQ derivatives with activity against *Mycobacterium tuberculosis*.

Karad et al. (2015) further evaluated the primary *in vitro* antituberculosis activity of fluorinated hexahydroquinoline derivatives (8a–8p) against *Mycobacterium tuberculosis* H37Rv using the Lowenstein–Jensen medium at a concentration of 250 μg/mL, with isoniazid and rifampicin serving as standard drugs. Compounds **8e, 8k,** and **8m** (Figure 12) showed strong activity with 94%, 95%, and 91% inhibition, respectively. Compounds 8L and 8p exhibited moderate inhibition, while the remaining derivatives showed weak or negligible activity against the tested strain.

### 3.8 Antischistosomal activity

**FIGURE 13:**
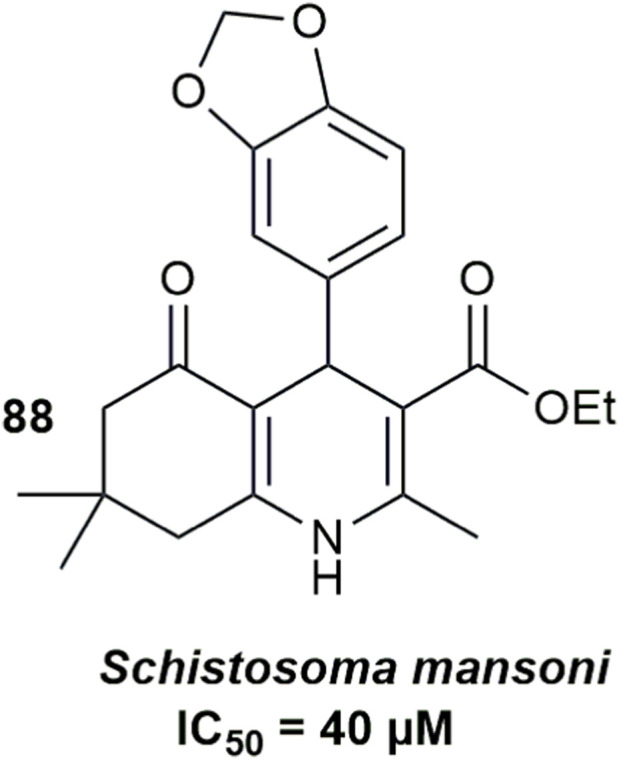
HHQ derivative with antischistosomal activity.

Oliveira et al. (2026) investigated the antischistosomal activity of a library of 1,4-dihydropyridines, including sixteen hexahydroquinolines (HHQs) and eight Hantzsch esters, using *in vitro* assays against adult *Schistosoma mansoni* worms. Following initial screening, active compounds with >50% inhibition at 50 µM were subjected to dose–response studies to determine IC_50_ values over 24–72 h, alongside cytotoxicity and selectivity evaluation in Vero cells. The HHQs exhibited moderate antischistosomal activity, with compound **88** (Figure 13) emerging as the most active (IC_50_ = 40.0 µM). However, their potency and selectivity were consistently inferior to those of the non-HHQ Hantzsch esters which showed low-micromolar IC_50_ values (13–15 μM at 24 h) and the highest selectivity indices.

## 4 Conclusion

Functionalized hexahydroquinolines (HHQs) represent a versatile class of heterocycles with significant promise in drug discovery. Advances in green, catalyzed and sustainable synthetic methods have enabled efficient access to diverse HHQ derivatives under environmentally friendly conditions. Biological evaluations consistently highlight their potential as antimalarial, anticancer, antioxidant, and antimicrobial agents, with particular relevance in overcoming multidrug resistance and targeting *Plasmodium falciparum* gametocytes. Despite these advances, further mechanistic studies, structure–activity relationship analyzes, and preclinical validations are required to translate HHQs into clinically viable therapeutics. Collectively, current evidence positions HHQs as emerging scaffolds of high value in medicinal chemistry and modern drug development.

## References

[B1] Abdelmoniem A. M. Mohamed M. F. Abdelmoniem D. M. Ghozlan S. A. S. Abdelhamid I. A. (2019). “Recent synthetic approaches and biological evaluations of amino Hexahydro-quinolines and their Spirocyclic Struct.,” 685(133242), pp. 875–915. 10.1016/j.colsurfa.2024.133242 30706793

[B2] Abou-Gharbia M. Abou-Gharbia M. (1986). Synthesis of novel hexahydroquinolines and hexahydroacridines. Heterocycles 24 (5), 1347–1353. 10.3987/r-1986-05-1347

[B3] Akbarpoor T. Khazaei A. Seyf J. Y. Sarmasti N. Gilan M. M. (2020). One-pot synthesis of 2-amino-3-cyanopyridines and hexahydroquinolines using eggshell-based nano-magnetic solid acid catalyst *via* anomeric-based oxidation. Res. Chem. Intermed. 46 (2), 1539–1554. 10.1007/s11164-019-04049-y

[B4] Albov D. V. Rybakov V. B. Babaev E. V. Aslanov L. A. (2004). X-ray mapping in heterocyclic design: XIII. Structure of substituted tetrahydroquinolines. Crystallogr. Rep. 49 (3), 430–436. 10.1134/1.1756641

[B5] Andalibi Salem S. Khazaei A. Seyf J. Y. Sarmasti N. Mahmoudiani Gilan M. (2021). Preparation of magnetic Cu(II) nano-structure (based on Nano-Fe3O4) and application to the synthesis of hexahydroquinoline derivatives. Polycycl. Aromat. Compd. 41 (2), 319–332. 10.1080/10406638.2019.1582076

[B6] Antaki H. (1963). The synthesis of ethyl 4:-aryl-5,6,7,8-tetrahydro-5-oxoquinoline-3- carboxylates and their derivatives. J. Chem. Soc. (Resumed), 4933–4938. 10.1039/jr9630004877

[B7] Çetin G. Çeti̇n B. Çolak B. Aşan M. Demi̇rel G. B. (2022). A new perspective for biological activities of novel hexahydroquinoline derivatives. J. Res. Pharm. 26 (1), 219–230. 10.29228/JRP.120

[B8] Choudhury S. K. Rout P. Parida B. (2017). “Metal-free activation of C (sp 3)– H bond, and a practical and rapid synthesis of privileged 1-Substituted 1. 2. 4 pp. 5275–5292. 10.1002/ejoc.201700471

[B9] Cosma C. Maia C. Khan N. Infantino M. Del Riccio M. (2024). “Leishmaniasis in humans and animals: a one health approach for surveillance, prevention and control in a changing world” Trop. Med. Infect. Dis., 9(11), p. 258. 10.3390/tropicalmed9110258 39591264 PMC11598728

[B10] Davydenko O. O. (2015). The synthesis and the antitubercular activity of 1-benzyl-4-hydroxy-2-oxo-1,2,5,6,7,8-hexahydroquinoline-3-carboxamides. J. Org. Pharm. Chem. 13 (3), 32–37. 10.24959/ophcj.15.851

[B11] Devi L. Robert A. R. Ganja H. Maddila S. Jonnalagadda S. B. (2020). A rapid, sustainable and environmental friendly protocol for the catalyst-free synthesis of 2-methyl-5-oxo-hexahydroquinoline-3-carboxylate *via* ultrasonic irradiation. Chem. Data Collect. 28, 100432. 10.1016/j.cdc.2020.100432

[B12] Dey S. Basak P. Ghosh P. (2020). A green synthetic approach towards one pot multi component synthesis of hexahydroquinoline and 9-Arylhexahydroacridine-1,8-dione derivatives catalyzed by sulphonated rice husk. ChemistrySelect 5 (48), 15209–15217. 10.1002/slct.202004121

[B13] Diagana T. Jones C. (2017). Hitting malaria where it hurts. Nat. Microbiol. 2 (10), 1336–1337. 10.1038/s41564-017-0036-z 29046526

[B14] Elebiju O. F. Ajani O. O. Oduselu G. O. Ogunnupebi T. A. Adebiyi E. (2023). Recent advances in functionalized quinoline scaffolds and hybrids — exceptional pharmacophore in therapeutic medicine. Front. Chem. 10 (1074331), 1–18. 10.3389/fchem.2022.1074331 36688036 PMC9859673

[B15] Faidallah H. M. Al-Juaid S. S. (2014). “Fast and green synthesis of 3-Cyano-8-Methyl-2-Oxo-4-Substituted 1, 2, 5, 6, 7, 8-Hexahydroquinolines,” Chem. Technol. Fuels Oils, 50(3), pp. 240–247. 10.1007/s10553-014-0516-2

[B16] Farhab M. Aziz M. W. Shaukat A. Cao M. X. Hou Z. Huang S. Y. (2025). Review of toxoplasmosis: what we still need to Do. Veterinary Sci. 12 (8), 772. 10.3390/vetsci12080772 40872723 PMC12390377

[B17] Fedoseev S. V. Lipin K. V. Ershov O. V. (2022). Synthesis and antiproliferative activity of 2-oxo-1,2-dihydropyridine-3,4-dicarbonitriles. Pharm. Chem. J. 56 (3), 325–328. 10.1007/s11094-022-02638-7

[B18] Ghiassi S. Mokhtary M. Sedaghat S. Kefayati H. (2019). Preparation, and antibacterial activity of chloroacetic acid immobilized on chitosan coated iron oxide decorated silver nanoparticles as an efficient catalyst for the synthesis of Hexahydroquinoline-3-Carboxamides. J. Inorg. Organomet. Polym. Mater. 29 (6), 1972–1982. 10.1007/s10904-019-01156-6

[B19] Ghorbani M. Noura S. Oftadeh M. Narimani M. Behbodi K. (2015). Preparation, characterization and application of novel ionic liquid as an efficient and reusable catalyst for the solvent-free synthesis of hexahydroquinolines. J. Mol. Liq. 209 (1), 224–232. 10.1016/j.molliq.2015.06.011

[B20] Girault S. Grellier P. Berecibar A. Maes L. Mouray E. Lemiere P. (2000). Antimalarial, antitrypanosomal, and antileishmanial activities and cytotoxicity of Bis(9-amino-6-chloro-2-methoxyacridines). Influ. Link. 6 (2), 2646–2654. 10.1021/jm990946n 10893302

[B21] Goudarziafshar H. Zafari M. Moosavi-zare A. R. (2024). Porous carbon/Fe 3 O 4 nanocomposite as a new preparation of polyhydroquinolines. RSC Adv. 14, 27565–27574. 10.1039/d4ra05432f 39221127 PMC11363064

[B22] Gupta V. Misra U. (2008). Synthesis and cardiovascular activity of difluoro-substituted hexahydroquinoline. Med. Chem. Res. 17 (2–7), 437–444. 10.1007/s00044-007-9078-8

[B23] Horn K. S. V. Zhao Y. Parvatkar P. T. Maier J. Mutka T. Lacrue A. (2024). Optimization of diastereomeric dihydropyridines as antimalarials. Eur. J. Med. Chem. 275, 116599. 10.1016/j.ejmech.2024.116599 38909569 PMC13047510

[B24] Kabir E. Uzzaman M. (2022). A review on biological and medicinal impact of heterocyclic compounds. Results Chem. 4 (August), 100606. 10.1016/j.rechem.2022.100606

[B25] Karad S. C. Purohit V. B. Raval D. K. Kalaria P. N. Avalani J. R. Thakor P. (2015). Green synthesis and pharmacological screening of polyhydroquinoline derivatives bearing a fluorinated 5-aryloxypyrazole nucleus. RSC Adv. 5 (21), 16000–16009. 10.1039/c5ra00388a

[B26] Kaushik A. Makarani N. Bharadava K. Gehlot J. Naik B. V. Singh A. (2025). “Antiprotozoal agents – integration of drug discovery, medicinal chemistry, and advanced computational approaches: an in-depth review,” Microbe, p. 7, 100395. 10.1016/j.microb.2025.100395

[B27] Khairullah A. R. Kurniawan S. C. Widodo A. Effendi M. H. Hasib A. Silaen O. S. M. (2024). A comprehensive review of toxoplasmosis: serious threat to human health. Open Public Health J. 17, 1–15. 10.2174/0118749445281387240202094637

[B28] Khan B. Hameed A. Minhaz A. Shah M. R. (2018). Synthesis and characterisation of calix[4]arene based bis(triazole)-bis(hexahydroquinoline): probing highly selective fluorescence quenching towards Mercury (Hg2+) analyte. J. Hazard. Mater. 347 (October 2017), 349–358. 10.1016/j.jhazmat.2018.01.022 29335217

[B29] Khazaei A. Sarmasti N. Seyf J. Y. Tavasoli M. (2015). Synthesis of hexahydroquinoline (HHQ) derivatives using ZrOCl2·8H2O as a potential green catalyst and optimization of reaction conditions using design of experiment (DOE). RSC Adv. 5 (123), 101268–101275. 10.1039/c5ra16102a

[B30] Khazaei A. Mahmoudiani Gilan M. Sarmasti N. (2017). Magnetic ‐ based picolinaldehyde – melamine copper complex for the one ‐ pot synthesis of hexahydroquinolines *via* hantzsch four ‐ component reactions. Appl. Organomet. Chem. 32 (August), 1–12. 10.1002/aoc.4151

[B31] Khazaei A. Sarmasti N. Yousefi Seyf J. (2018). Anchoring high density sulfonic acid based ionic liquid on the magnetic nano-magnetite (Fe3O4), application to the synthesis of hexahydroquinoline derivatives. J. Mol. Liq. 262 (2017), 484–494. 10.1016/j.molliq.2018.04.125

[B32] Kismetli E. Safak C. Erol K. Sirmagül B. Linden A. (2004). Studies on 3-diethylaminocarbonyl-1,4,5,6,7,8-hexahydroquinoline derivatives and their calcium channel antagonistic activities *in vitro* . Arzneimittel-Forschung/Drug Res. 54 (7), 371–375. 10.1055/s-0031-1296986 15344840

[B33] Lak P. Amini M. Safavi M. Shafiee A. Shahverdi A. R. (2008). Enhancement of the antibacterial activity of ciprofloxacin against *Staphylococcus aureus* by 3-alkyl esters and 3-aryl esters of hexahydroquinoline derivatives. Arzneimittel-Forschung/Drug Res. 58 (9), 464–468. 10.1055/s-0031-1296540 18972877

[B34] Lee S. Kim M. S. Hayat F. Shin D. (2019). Recent advances in the discovery of novel antiprotozoal agents. Molecules 24 (21), 3886. 10.3390/molecules24213886 31661934 PMC6864685

[B35] Liu S. Cai M. Liu Z. Gao W. Li J. Li Y. (2024). “Comprehensive insights into the development of Anti-toxoplasmosis drugs: current advances, obstacles and,” J. Med. Chem., 67(23), 20740–20764. 10.1021/acs.jmedchem.4c01733 39589152

[B36] Loukhmi Z. Elmakssoudi A. Thoume A. Achagar R. Roby O. Dahib Z. (2024). “Synthesis, characterization and theoretical studies of hexahydroquinoline derivatives using nano pyrophosphate as an effective renewable catalyst,” Colloids Surfaces A Physicochem. Eng. Aspects 685 133242. 10.1016/j.colsurfa.2024.13342

[B37] Majoor A. Michel G. Marty P. Boyer L. Pomares C. (2025). “Leishmaniases: strategies in treatment development,” Parasite, 32, 18. 10.1051/parasite/2025009 40043198 PMC11882135

[B38] Makarani N. Bharadava K. Kaushik A. Dave A. Gangawane A. K. Kaushal R. S. (2025). Leishmaniasis: a multifaceted approach to diagnosis, maladies, drug repurposing and way forward. Microbe 6 (January), 100239. 10.1016/j.microb.2025.100239

[B39] Maleki A. Akbarzade A. R. Bhat A. R. (2017). Green synthesis of polyhydroquinolines *via* MCR using Fe_3_O_4_/SiO_2_ -OSO_3_H nanostructure catalyst and prediction of their pharmacological and biological activities by PASS. J. Nanostructure Chem. 7 (4), 309–316. 10.1007/s40097-017-0240-7

[B40] Mann S. Frasca K. Scherrer S. Henao-Martínez A. F. Newman S. Ramanan P. (2021). “A review of leishmaniasis: current knowledge and future directions,” Curr. Trop. Med. Rep., 8, pp. 121–132. 10.1007/s40475-021-00232-7 33747716 PMC7966913

[B41] Maya D. J. Morello A. Repetto Y. Tellez R. Rodriguez A. Zelada U. (2000). “Effects of 3-chloro-phenyl-1, 4-dihydropyridine derivatives on trypanosome cruzi epimastigotes,” Comp. Biochem. Physiology Part C Pharmacol. Toxicol. Endocrinol., 125(1), 103–109. 10.1016/s0742-8413(99)00096-1 11790334

[B42] Mielcarek J. Safak C. Simsek R. Matłoka A. (2002). Photochemical study of hexahydroquinoline derivatives - a new group of calcium antagonists. Arch. Pharm. 335 (2), 77–82. 10.1002/1521-4184(200203)335:2/3<77::AID-ARDP77>3.0 12007110

[B43] Mielcarek J. Matłoka A. Grobelny P. (2005). Identification of photoproducts of hexahydroquinoline derivatives by GC-EI-MS and HPLC-ESI-MS. Drug Dev. Industrial Pharm. 31 (9), 861–869. 10.1080/03639040500271852 16305997

[B47] Mirani Nezhad S. Nazarzadeh Zare E. Davarpanah A. Pourmousavi S. A. Ashrafizadeh M. Kumar A. P. (2022). “Ionic liquid-assisted fabrication of bioactive heterogeneous magnetic nanocatalyst with antioxidant and antibacterial activities for the synthesis of polyhydroquinoline derivatives.” 10.3390/molecules27051748 PMC8912081 35268849

[B44] Molaei S. Farhadi G. Talezari M. Gholizadeh N. Mahnam K. Keivanloo A. (2023). Triazole hybrids in deep eutectic solvent as anti-leishmanial agents and molecular modeling studies. J. Biomol. Struct. Dyn. 0 (0), 1–17. 10.1080/07391102.2023.2224897 37325813

[B45] Morbale S. T. Shinde S. S. Jadhav S. D. Deshmukh M. B. Patil S. S. (2015). Modified eggshell catalyzed, one-pot synthesis and antimicrobial evaluation of 1, 4-dihydropyridines and polyhydroquinolines. Lett 7 (12), 169–182.

[B46] Moshtaghi A. Somaieh Z. Davood O. (2016). Ammonium acetate as a catalyst and/or reactant in the reaction of dimedone, aromatic aldehyde, and malononitrile: synthesis of tetrahydrobenzo [ b ] pyrans and hexahydroquinolines. Monatsh. für Chem. - Chem. Mon., 147 0–5. 10.1007/s00706-016-1683-0

[B48] Oduselu G. O. Elebiju O. F. Ogunnupebi T. A. Akash S. Ajani O. O. Adebiyi E. (2024). Employing hexahydroquinolines as PfCDPK4 inhibitors to combat malaria transmission: an advanced computational approach. Adv. Appl. Bioinforma. Chem. 17 (September), 83–105. 10.2147/AABC.S476404 39345873 PMC11430315

[B49] Oliveira T. A. S. Silva J. B. A. Silva N. B. S. Félix P. C. A. Dos Santos D. A. de Oliveira A. M. (2024). “Antibacterial and antileishmanial activity of 1, 4-Dihydropyridine derivatives,” Chem. and Biodivers., 22(1), p. e202401300. 10.1002/cbdv.202401300 39231212

[B50] Oliveira T. A. S. Robles Y. R. Al Nasr I. S. Koko W. S. Khan T. A. Daoud I. (2025). “Antileishmanial and antitoxoplasmal activities of 1, 4- dihydropyridines,” ACS Omega, 10(28), pp. 31066–31076. 10.1021/acsomega.5c04551 40727813 PMC12290673

[B51] Oliveira T. A. S. Zago M. H. Maciel L. G. Robles Y. R. Magalhães L. G. Crotti A. E. (2026). Antischistosomal activity of 1, 4-Dihydropyridines, 1–15.

[B52] Pareyn M. Alves F. Burza S. Chakravarty J. Alvar J. Diro E. (2025). Leishmaniasis. Nat. Rev. Dis. Prim. 11, 81. 10.1038/s41572-025-00663-w 41266459

[B53] Patil D. Chandam D. Mulik A. Jagdale S. Patil P. Deshmukh M. (2017). One pot four component sequential synthesis of hexahydroquinoline derivatives in aqueous media *via* enaminone intermediates: a green protocol. J. Saudi Chem. Soc. 21, S329–S338. 10.1016/j.jscs.2014.04.001

[B54] Pehli̇Vanlar E. Çakir D. A. Sanajou S. Tezel Yalçin H. Baydar T. Erkekoğlu P. (2024). Synthesis and characterization of new hexahydroquinoline derivatives and evaluation of their cytotoxicity, intracellular ROS production, and inhibitory effects on inflammatory mediators. Turkish J. Chem. 48 (4), 659–675. 10.55730/1300-0527.3686 39296790 PMC11407359

[B55] Popandova-Yambolieva K. (1990). “Synthesis of ethyl 5-Aryl-2-oxo-7-phenyl-1, 2, 3, 4, 4a, 5-hexahydroquinoline-4a-carboxylates,” Synthesis, 1(01), pp. 84–86. 10.1055/s-1990-26796

[B56] Pourmand M. R. Yousefi M. Salami S. A. Amini M. (2014). Evaluation of expression of NorA efflux pump in ciprofloxacin resistant Staphylococcus aureus against hexahydroquinoline derivative by real-time PCR. Acta Medica Iran. 52 (6), 424–429. 25130148

[B57] Ranjbar S. Edraki N. Firuzi O. Khoshneviszadeh M. Miri R. (2019). 5-Oxo-hexahydroquinoline: an attractive scaffold with diverse biological activities. Mol. Divers. 23 (2), 471–508. 10.1007/s11030-018-9886-4 30390186

[B58] Ranjbar S. Khonkarn R. Moreno A. Baubichon-Cortay H. Miri R. Khoshneviszadeh M. (2019). 5-Oxo-hexahydroquinoline derivatives as modulators of P-gp, MRP1 and BCRP transporters to overcome multidrug resistance in cancer cells. Toxicol. Appl. Pharmacol. 362, 136–149. 10.1016/j.taap.2018.10.025 30391378

[B59] Rizwan H. M. Abbas H. Sajid M. S. Maqbool M. Jones M. K. Ullah M. I. (2021). “Drug resistance in protozoal infections,” in Biochemistry of drug resistance *,*. Springer International Publishing, Cham, pp. 95–142. 10.1007/978-3-030-76320-6_4

[B60] Rose U. Drager M. (1992). K 8644. J. Med. Chem. 35 (6), 2238–2243. 10.1021/jm00090a014 1319494

[B61] Rostamnia S. Nuri A. Xin H. Pourjavadi A. Hosseini S. H. (2013). Water dispersed magnetic nanoparticles (H_2_O-DMNPs) of γ-Fe_2_O_3_ for multicomponent coupling reactions: a green, single-pot technique for the synthesis of tetrahydro-4H-chromenes and hexahydroquinoline carboxylates. Tetrahedron Lett. 54 (26), 3344–3347. 10.1016/j.tetlet.2013.04.048

[B62] Shahraki O. Khoshneviszadeh M. Dehghani M. Mohabbati M. Tavakkoli M. Saso L. (2020). xo-hexahydroquinoline derivatives and their tetrahydroquinoline counterparts as multidrug resistance reversal agents. Molecules, 25 (8), 1839. 10.3390/molecules25081839 32316291 PMC7221826

[B63] Sharifabad S. S. Mirjalili B. B. F. Bamoniri A. (2022). Nano-SiO2/Taurine as a new natural based catalyst for synthesis of hexahydroquinolines derivative. Iran. J. Catal. 12 (3).

[B64] Şimşek R. Oztürk G. S. Vural I. M. Gündüz M. G. Sarioglu Y. Safak C. (2008). Synthesis and calcium modulatory activity of 3-alkyloxy-carbonyl-4- (disubstituted)aryl-5-oxo-1,4,5,6,7,8-hexahydroquinoline derivatives. Arch. Pharm. 341 (1), 55–60. 10.1002/ardp.200700087 17994603

[B65] Smirnova T. A. Gavrilov M. Y. Vasilyuk M. V. Zaks A. S. Kon'shin M. E. (1999). Synthesis and antiinflammatory and analgesic activity of 2-oxo-1,2,5,6,7,8-hexahydroquinoline-4-carboxylic acid amides. Pharm. Chem. J. 33 (1), 27–28. 10.1007/BF02508413

[B66] Sridharan V. Suryavanshi P. A. Menendez J. C. (2011). “Advances in the chemistry of tetrahydroquinolines,” Chem. Reviews, 111(11), pp. 7157–7259. 10.1021/cr100307m 21830756

[B67] Srivastava S. (2024). The significance of heterocyclic compounds in biological activity and medicinal chemistry: a review study. Int. J. Nov. Res. Dev. 9 (12), 339–355.

[B68] Stankevich E. I. Grinshtein E. E. Dubur G. Y. (1975). Structures of the products of the reaction of β-aminovinylcarbonyl compounds, a β-diketone, and an aldehyde. Chem. Heterocycl. Compd. 11 (2), 196–198. 10.1007/BF00471397

[B69] Ukrainets I. V. Kolesnik E. V. Sidorenko L. V. Gorokhova O. V. Turov A. V. (2006). 4-hydroxy-2-quinolones. 95. Synthesis, structure, and antitubercular properties of hetarylamides of 4-hydroxy-2-oxo-1,2,5,6,7,8-hexahydroquinoline-3- carboxylic acid. Chem. Heterocycl. Compd. 42 (6), 765–775. 10.1007/s10593-006-0159-2

[B70] Vanaerschot M. Lucantoni L. Li T. Combrinck J. M. Ruecker A. Kumar T. R. S. (2017). Hexahydroquinolines are antimalarial candidates with potent blood-stage and transmission-blocking activity. Nat. Microbiol. 2 (10), 1403–1414. 10.1038/s41564-017-0007-4 28808258 PMC5708124

[B71] Veale C. Müller R. (2020). Recent highlights in anti-infective medicinal chemistry from South Africa. ChemMedChem 15 (10), 809–826. 10.1002/cmdc.202000086 32149446

[B72] Wu R. Zhou H. Y. Song J. F. Xia Q. H. Hu W. Mou X. D. (2021). Chemotherapeutics for Toxoplasma gondii: molecular biotargets, binding modes, and structure − activity relationship investigations. J. Med. Chem. 64, 17627–17655. 10.1021/acs.jmedchem.1c01569 34894691

[B73] Zahedi M. Asgari Q. Badakhshan F. Sakhteman A. Ranjbar S. Khoshneviszadeh M. (2020). Anti- Toxoplasma gondii activity of 5-oxo-hexahydroquinoline derivatives: synthesis, *in vitro* and *in vivo* evaluations, and molecular docking analysis. Res. Pharm. Sci. 15 (August), 367–380. 10.4103/1735-5362.293515 33312215 PMC7714012

[B74] Zarghi A. Sabakhi I. Topuzyan V. Hajimahdi Z. Daraie B. (2014). Hexahydroquinoline derivatives as selective Cyclooxygenase-2 inhibitors. Iran. J. Pharm. Res. 13, 61–69. 24711830 PMC3977054

[B75] Zonouz A. M. Abedinpour S. (2025). “A review on the methods of preparing 1, 4-dihydropyridine derivatives,” 2(2), pp. 56–84. 10.22049/cic.2024.29549.1038

