# Correction: Emerging insights into chemistry and therapeutic potentials of functionalized hexahydroquinolines

**DOI:** 10.3389/fchem.2026.1852717

**Published:** 2026-05-01

**Authors:** Gbolahan O. Oduselu, Rhoda O. Olatuyi, Omowunmi O. Fatoki, Damilola S. Bodun, Promise E. Sunday, Wellington Oyibo, Olayinka O. Ajani

**Affiliations:** 1 West African Center for Cell Biology of Infectious Pathogens (WACCBIP), College of Basic and Applied Sciences, University of Ghana, Accra, Ghana; 2 Department of Chemistry, College of Science and Technology, Covenant University, Ota, Nigeria; 3 Department of Pharmaceutical and Medicinal Chemistry, University of Uyo, Uyo, Nigeria; 4 ANDI Center of Excellence for Malaria Diagnosis, College of Medicine, University of Lagos, Lagos, Nigeria

**Keywords:** antiprotozoal agents, drug discovery, heterocyclic compounds, pharmacological potential, quinoline

There was a mistake in Table 1, Scheme 2 as published. One of the key reagents mentioned in table (S/N 2) as part of the Antaki synthesis of hexahydroquinoline derivatives was wrongly written as “Cyclohexane-1,2-dione”. The corrected Table 1, Scheme 2 appear below.

**TABLE 1:** Synthetic strategies for structurally dominant hexahydroquinoline derivatives.

S/N	Category	Method/Catalyst	Key reagents	Conditions	Time	Yield	Unique advantage
1	Classical (Çetin et al., 2022)	Hantzsch-type	4,4-dimethylcyclohexane-1,3-dione, acetoacetate, disubstituted benzalaldehyde, ammonium acetate	Refluxed in methanol, one-pot	8 h	69%–76%	Simple multicomponent foundation method
2	Classical (Antaki, 1963)	Antaki synthesis (modified Hantzsch-type)	Cyclohexane-1,3-dione, ethyl β-aminocrotonate, nitrobenzaldehyde	EtOH + acetic acid, reflux	1 h	–	First isolated HHQs as intermediates
3	Classical (Stankevich, Grinshtein and Dubur, 1975)	Stankevich Synthesis (modified Hantzsch-type)	Dimedone, ethyl β-aminocrotonate, paraformaldehyde	Reflux in EtOH	1 h	37.8%	Straightforward, forms crystalline HHQs
4	Classical (modified) Abou-Gharbia (1986)	Modified Stankevich Synthesis	Dimedone, ethyl 3-aminocrotonate, heterocyclic aldehydes (N-methylimidazole-2-carbox- aldehyde and thiazole-2- carboxaldehyde)	Reflux in EtOH	12 h	37%–72%	Enhances structural diversity
5	Classical (modified) Rose and Drager (1992)	Modified Stankevich Synthesis	Dimedone, acetoacetate ester, benzaldehyde	Ammonia, conc. Acetic acid heat, metallic sodium in methanol	2.5 h	15%–19%	Produces chiral HHQ precursors, the bulky chiral groups were removed with metallic sodium in methanol
6	One-Pot/MCR (Şimşek et al., 2008)	Modified Hantzsch-type	4,4- or 5,5-dimethyl-1,3-cyclohexanedione aldehyde, methyl (or ethyl) aminocrotonate, aromatic aldehyde	One-pot MCR, refluxed in methanol	4 h	63%–81%	Fast one-pot multicomponent carboxamide formation
7	Green (Catalyzed) Rostamnia et al. (2013)	Water dispersed γ-Fe_2_O_3_ nanoparticles	Aromatic aldehyde, dimedone, β-dicarbonyl compound, ammonium acetate	Water, 10 mol% γ-Fe_2_O_3_ catalyst	2.5–3 h	90%–96%	Water-based, reusable catalyst
8	Green (Catalyzed) Salem et al. (2021)	Cu(II) nanomagnetic catalyst, (Fe_3_O_4_@SiO_2_@Si-(CH_2_)_3_@HMTA@Cu(II))	Aldehyde, dimedone, acetoacetate	Solvent-free	3–15 min	50%–96%	Very fast, high efficiency, catalyst separable from reaction mixture with ethyl acetate
9	Green (Natural Catalyst) Ghiassi et al. (2019)	Verjuice (unripe grape juice)	ArylAldehyde, dimedone, acetoacetanilide, ammonium acetate	EtOH, 70 °C, natural acid catalyst	20 min	86%–96%	Biocompatible acidic catalyst, milder conditions
10	Green (Catalyzed) Moshtaghi, Somaieh and Davood (2016)	Imidazole catalyst	Nitrobenzaldehyde, malononitrile, 3-aminodimedone	EtOH reflux + imidazole	45 min	76%	Cuts reaction time drastically relative to the non-catalyzed synthesis
11	Green (Catalyzed, statistically optimized) (Khazaei et al., 2015)	ZrOCl_2_·8H_2_O Lewis acid catalyst with CCD optimization	Aldehyde, dimedone, β-dicarbonyl, ammonium acetate	Solvent-free, 85 °C, 0.15 M catalyst	40–180 s	79%–98%	Ultrafast HHQ formation guided by Central Composite Design
12	Green (Catalyzed, statistically optimized) Khazaei et al. (2017)	Fe_3_O_4_@SiO_2_-supported Cu complex with CCD optimization	Aldehyde, dimedone, β-dicarbonyl, ammonium acetate	Solvent-free, 88 °C	2–17 min	71%–92%	Strong Lewis acid activation with magnetic recyclability
13	Green (Catalyzed, statistically optimized) Khazaei et al. (2018)	SO_3_H-functionalized - Fe_3_O_4_supported Brønsted acidic ionic liquid, with CCD optimization	Aldehyde, dimedone, β-dicarbonyl, ammonium acetate	Solvent-free, 65 °C	4–11 min	48%–90%	Recyclable, magnetic recovery
14	Green (Bionanocomposite) Maleki (2017)	γ-Fe_2_O_3_/Cu@cellulose	Aromatic Aldehyde, dimedone, ethyl acetoacetate	Solvent-free, 3 mg catalyst	9–35 min	80%–98%	Highly reusable catalyst
15	Green (Metal Nanocatalyst) Ghorbani et al. (2015)	Pd-Schiff base on Fe_3_O_4_	Aromatic Aldehyde, dimedone, ethyl acetoacetate	EtOH:H_2_O (1:1), 75 °C	6–12 min	83%–94%	High turnover, recyclable (magnetically recovered), short reaction time
15	Green (Bio-derived catalyst) (Sharifabad et al., 2022)	Nano-SiO_2_/Taurine solid acid catalyst	Aldehyde, dimedone, acetoacetate, ammonium acetate	Solvent-free, 80 °C–85 °C	10–35 min	−86%–96%	Bio-derived taurine catalyst; stable, reusable
16	Green (Nanostructured Inorganic catalyst) Loukhmi et al. (2024)	Nano-Na_2_CaP_2_O_7_ diphosphate catalyst	Aldehydes, malononitrile or ethyl cyanoacetate, dimedone, ammonium acetate	EtOH, 80 °C	≤10 min	62%–97%	Rapid one-pot synthesis using recyclable nanopyrophosphate catalyst
17	Green (Biowaste Catalyst) Dey, Basak and Ghosh (2020)	Sulfonated rice husk (SRH) (2020)	Malononitrile, dimedone, 1-phenylethanone	Solvent-free, 70 °C, 60 mg SRH	20 min	Up to 96%	Cheap, biodegradable, highly reusable
18	Green (Biowaste Catalyst) Akbarpoor et al. (2020)	Eggshell-derived Fe_3_O_4_@Ca(HSO_4_)_2_catalyst	Aromatic aldehyde, dimedone, acetoacetate	Solvent-free, 71 °C	3–9 min	68%–98%	Converts waste to high-performing catalyst
19	Green (Biowaste Catalyst) Morbale et al. (2015)	Modified eggshells (MES)	Aromatic aldehyde, dimedone, acetoacetate	EtOH:H_2_O (1:1), 80 °C	45–70 min	83%–91%	Cheap, sustainable catalyst
20	Green (Biowaste Nanocatalyst) (Goudarziafshar et al., 2024)	Peanut shell–derived porous carbon/Fe_3_O_4_ nanocomposite	Aryl aldehydes, dimedone, ethyl acetoacetate, ammonium acetate	Solvent-free, 75 °C, 3 mg catalyst	6–22 min	81%–96%	Biomass-derived, magnetically recoverable nanocatalyst; very low catalyst loading
21	Green (Catalyst-free) Patil et al. (2017)	No catalyst	Dimedone, ammonium acetate, aromatic aldehyde, malononitrile	Water only	–	–	Fully catalyst-free green synthesis
22	Green (Catalyst-Free) Devi et al. (2020)	Ultrasonication assisted synthesis	Dimedone, benzaldehydes, benzyl acetoacetate, and ammonium acetate	Ethanol, ultrasonic irradiation at room temperature	10 min	92.98%	One-pot multicomponent,non-catalyzed reaction

**SCHEME 2:**
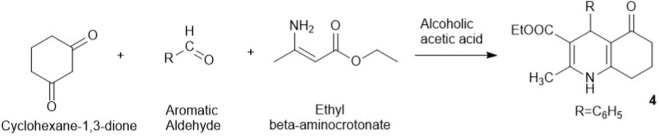
Antaki’s synthesis of HHQ derivatives.

The original article has been updated.

